# Capturing tumour heterogeneity in pre- and post-chemotherapy colorectal cancer ascites-derived cells using single-cell RNA-sequencing

**DOI:** 10.1042/BSR20212093

**Published:** 2021-12-07

**Authors:** Tiraput Poonpanichakul, Meng-Shin Shiao, Natnicha Jiravejchakul, Ponpan Matangkasombut, Ekaphop Sirachainan, Varodom Charoensawan, Natini Jinawath

**Affiliations:** 1 Chakri Naruebodindra Medical Institute, Faculty of Medicine Ramathibodi Hospital, Mahidol University, Samut Prakan, Thailand; 2 Department of Microbiology, Faculty of Science, Mahidol University, Bangkok, Thailand; 3 Systems Biology of Diseases Research Unit (SyBiD), Faculty of Science, Mahidol University, Bangkok, Thailand; 4 Research Center, Faculty of Medicine Ramathibodi Hospital, Mahidol University, Bangkok, Thailand; 5 Division of Medical Oncology, Department of Internal Medicine, Faculty of Medicine Ramathibodi Hospital, Mahidol University, Bangkok, Thailand; 6 Ramathibodi Comprehensive Cancer Center, Faculty of Medicine Ramathibodi Hospital, Mahidol University, Bangkok, Thailand; 7 Department of Biochemistry, Faculty of Science, Mahidol University, Bangkok, Thailand; 8 Integrative Computational BioScience (ICBS) Center, Mahidol University, Nakhon Pathom, Thailand; 9 Program in Translational Medicine, Faculty of Medicine Ramathibodi Hospital, Mahidol University, Bangkok, Thailand

**Keywords:** chemotherapy, colorectal cancer, malignant ascites, scRNA-seq, tumour heterogeneity

## Abstract

Malignant ascites is an abnormal accumulation of fluid within the peritoneal cavity, caused by metastasis of several types of cancers, including colorectal cancer (CRC). Cancer cells in ascites reflect poor prognosis and serve as a good specimen to study tumour heterogeneity, as they represent a collection of multiple metastatic sites in the peritoneum. In the present study, we have employed single-cell RNA-sequencing (scRNA-seq) to explore and characterise ascites-derived cells from a CRC patient. The samples were prepared using mechanical and enzymatic dissociations, and obtained before and after a chemotherapy treatment. Unbiased clustering of 19,653 cells from four samples reveals 14 subclusters with unique transcriptomic patterns in four major cell types: epithelial cells, myeloid cells, fibroblasts, and lymphocytes. Interestingly, the percentages of cells recovered from different cell types appeared to be influenced by the preparation protocols, with more than 90% reduction in the number of myeloid cells recovered by enzymatic preparation. Analysis of epithelial cell subpopulations unveiled only three out of eleven subpopulations with clear contraction after the treatment, suggesting that the majority of the heterogeneous ascites-derived cells were resistant to the treatment, potentially reflecting the poor treatment outcome observed in the patient. Overall, our study showcases highly heterogeneous cancer subpopulations at single-cell resolution, which respond differently to a particular chemotherapy treatment. All in all, this work highlights the potential benefit of single-cell analyses in planning appropriate treatments and real-time monitoring of therapeutic response in cancer patients through routinely discarded ascites samples.

## Introduction

Colorectal cancer (CRC) is one of the most common cancers globally. CRC exhibits high mortality rate [1] and high risk of metastasis [2]; it can progress and metastasise to several body sites, including the peritoneum. Approximately 7–26% of the CRC patients had peritoneal metastasis, resulting in malignant ascites, and this poses a poorer prognosis and higher risk of recurrence [3]. Metastasis and chemoresistance in cancer are often correlated [4,5]. However, it is challenging to accurately assess how the cancer cells respond to chemotherapy treatments and thus determine an appropriate regimen [6]. When the direct assessment of primary tumour cells is not practical, most clinical investigations rely on known blood markers to evaluate cancer status [7–9]. Alternatively, malignant ascites, which represents another biofluid source for liquid biopsy, can serve as an important biological material for molecular characterisation of solid tumours. It is readily available in large volume when cancer patients undergo intermittent abdominal paracentesis to relieve abdominal discomfort, which is part of symptomatic treatment. However, so far there are only a few studies that characterise the potential use of malignant ascites. Some of those studies aim to find biomarkers for cancer diagnosis [10,11] or study the molecular phenotype of ascites-derived cells [12]. Furthermore, ascites is gaining recognition as a unique form of tumour microenvironment responsible for cancer progression and treatment resistance. Since there are multiple cell types in malignant ascites including tumour cells, stromal cells and immune cells [13], the ability to simultaneously analyse each cellular population and subpopulation should help clarify the roles of ascites samples in cancer progression and its potential usage as a liquid biopsy specimen.

In the era of high-throughput molecular technologies such as massively parallel sequencing, transcriptomics has been intensively applied to study the gene expression characteristics of different types of cancers. One of the most comprehensive examples of high-throughput gene expression profiling of cancers is The Cancer Genome Atlas (TCGA) project (https://www.cancer.gov/tcga) and Consensus Molecular Subtype (CMS) classification systems [14]. Both demonstrate the benefits of harnessing the gene expression signatures and clinical features to classify patients based on treatment responses and the disease outcomes. However, overall progress is still largely hindered by the limitations of resolving intratumoural heterogeneity, and hence the majority of the expression profiles represent the ‘average’ molecular characteristics of highly heterogeneous cancer cells [15,16].

Single-cell RNA-sequencing (scRNA-seq) is a powerful tool that enables transcriptomic profiling of individual cancer cells, and empowers clinical implementation of more tailored treatments [17–19]. It has been proposed that characterisation of transcriptomic profiles of the CRC samples using scRNA-seq would be an important step to understand the carcinogenesis and progression mechanisms of this cancer [20,21], as well as to develop personalised treatment against it [17]. In the past 5 years, several studies have employed scRNA-seq to investigate the genomic heterogeneity of CRC in several aspects. Li and co-workers, for instance, investigated the intratumoural heterogeneity of CRC cells at primary site, as compared with adjacent normal mucosal tissues [22]. Dai and co-workers investigated the heterogeneity of CRC tissue at primary site [23]. Despite being a practical source of patient samples for biomolecular analysis, to the best of our knowledge, no study so far has described intratumoural heterogeneity of malignant ascites in CRC patients. Indeed, the promising prospect of using ascites-derived cells to investigate the cancer’s molecular profile was demonstrated by Tang-Huau and co-workers, who successfully utilised scRNA-seq to dissect cellular heterogeneity and myeloid cells cross-presentation in ovarian cancer [24].

Here, we characterised intratumoural heterogeneity from ascites-derived cancer cells using a droplet-based scRNA-seq method. To investigate whether different single cell dissociation methods may alter cell population size and gene expression, the samples were prepared using different cell preparation protocols: mechanistic or enzymatic dissociation. We observed intratumoural heterogeneity and population dynamic changes between a cycle of modified FOLFIRI (mFOLFIRI) chemotherapy regimen, which corresponded well to the clinical outcome observed in our patient. Taken together, we have provided evidence of how the single-cell technology can be employed to dissect molecular complexity of intratumoural heterogeneity, the key insight required to improve the accuracy of molecular markers and the efficacy of the treatments against cancers.

## Materials and methods

### Patient information and clinical diagnosis

A 62-year-old female patient with underlying hypertension presented with weight loss, constipation, and haematochezia. CT colonoscopy showed polypoid polyps at distal rectum. Sigmoidoscopy showed 50% circumferential mass at 5–15 cm from anal verge with partial obstruction. Pathology report of the biopsy sample showed moderately differentiated adenocarcinoma. Molecular study of the tumour showed KRAS codon 12 (G12C) mutation. CT scan of whole abdomen revealed two small (7 and 8 mm) hypodense lesions at hepatic segments VII and VIII, circumferential irregular enhancing wall of rectum 5.6 cm from anal verge, perirectal fat extension, and multiple perirectal lymphadenopathy. Chest CT scan showed multiple lung nodules (2–4 mm). The patient was diagnosed with advanced rectal cancer (cT3N2bM1) with lung and liver metastases.

### Clinical course and treatment history

A palliative chemotherapy, modified FOLFOX6 (mFOLFOX6), was started in November 2017. After the fourth cycle, MRI showed a decrease in size of liver nodule in segment VIII from 8 to 4 mm, and disappearance of segment VII nodule. The patient received two more cycles of mFOLFOX6, then requested to change the regimen due to intolerable side effects, and thus was switched to capecitabine/oxaliplatin (CapeOx). Due to thrombocytopenia and neuropathy, oxaliplatin dose was reduced and finally omitted. After the fourth cycle of CapeOx, she developed abdominal distension from massive ascites. CT scan showed peritoneal metastasis but rectal mass size was decreased and no liver nodule was found. Progressive disease was diagnosed. She underwent abdominal paracentesis. The ascites cytology showed adenocarcinoma. She then received a second-line palliative chemotherapy, mFOLFIRI, in May 2018. Ascitic fluid samples were collected before and after the first cycle of mFOLFIRI. Carcinoembryonic antigen (CEA) slightly changed from 6.8 to 6.4 ng/ml after the treatment. Later, the patient developed new pleural effusion after the second cycle of mFOLFIRI and required frequent thoracocentesis and abdominal paracentesis procedures. Bevacizumab was added to mFOLFIRI in the third cycle in July 2018. Finally, her performance status declined gradually, she could not receive any further palliative chemotherapy and best supportive care was given. All samples were obtained with informed consent after the approval from the Institutional Review Board at Faculty of Medicine Ramathibodi Hospital, Mahidol University under certificate number COA.MURA2018/1067. Detailed clinical timeline can be found in Supplementary Figure S1.

### Patient sample collection and single cell preparation

Approximately 500 ml of ascitic fluid was collected from the patient and was transferred to the laboratory for processing immediately. Ascites was pre-filtered by 70-μm cell strainers (Corning, cat. no. 431751, U.S.A.) with gentle mechanical motorisation using pipette tips to assist cell clumps to pass through filters. The filtered ascitic fluid was collected in 50-ml falcon tubes. Cells in the filtered ascites were then subjected to centrifugation at 100 rcf for 10 min at 25°C and the clear supernatant was carefully removed. Next, the sedimented cells were treated with the RBC lysis buffer (Qiagen, cat. no.158902, Germany) to remove the red blood cells (RBCs). One millilitre of pre-chilled RBC lysis buffer was gently mixed with the cells, and incubated at room temperature for 5–10 min depending on the observed amount of RBCs in the cell pellets. Ten millilitres of pre-chilled Dulbecco’s phosphate-buffered saline (DPBS, calcium- and magnesium-free) was later added and cells were again collected by centrifugation at 300 rcf for 10 min at 25°C. For mechanical dissociation, the cells were assessed again under the microscope; if many cell clumps were still visualised, another round of filtering with 70-μm cell strainers was applied. For enzymatic dissociation, after RBC removal, we treated the cells with 2 ml of Accumax (Innovative Cell Technologies, Inc., U.S.A.) and incubated the cells at 37°C for 10 min, after which the cells were quickly assessed under the microscope. If there were still many visible cell clumps, another 10–20-min incubation was applied. Accumax reaction was terminated by the addition of 10-ml fresh culture medium, followed by centrifugation at 300 rcf for 10 min at 25°C to collect cell pellets. Finally, viable cell numbers after the completion of both dissociation methods were assessed by haemocytometer using Trypan Blue. The single cells were then resuspended in 90% FBS+10% DMSO at a concentration of 10^7^ cells/ml per tube, kept in a slow-cooling freezing container at −80°C, and cryopreserved in the vapour phase of liquid nitrogen the next day for long-term storage.

### scRNA-seq library preparation

Frozen cells were thawed and processed according to the recommended protocol for human PBMCs (10x Genomics, U.S.A.). Cell quantity and viability were checked with the haemocytometer under the microscope. Dead Cell Removal Kit (Miltenyi Biotec, cat. no. 130-090-101, Germany) was applied according to the manufacturer’s protocol. Cells were resuspended in phosphate-buffered saline (PBS) supplemented with 0.04% bovine serum albumin (BSA) (Merck, cat. no. 12659, Germany) before undergoing single-cell preparation protocol using the Chromium Single Cell 3′ v2 (10x Genomics, cat. no. PN-120267, U.S.A.). scRNA-seq libraries were sequenced with the Illumina HiSeq platform by Macrogen Inc. (South Korea).

### Bioinformatics analyses

Sequenced reads were checked for overall sequencing qualities using FastQC [25], and then mapped, and unique molecular identifiers (UMIs) quantified using Cell Ranger version 3.0.1 (10x Genomics, U.S.A.), using 10x human genome GRCh38 version 1.2.0 as the reference. Seurat [26] package v3.1.0 was mainly used for further analysis, including discarding low-quality cells in the case that the number of expressed genes is less than 200 genes per cell, or the percentage of mitochondrial genes is higher than 20% in a cell. Genes that were detected in less than five cells were also removed. SoupX [27] was applied to regress out the ambient RNAs. Doublets were determined using DoubletFinder [28], and removed from further downstream analysis. Dimensionality reduction, principal component analysis (PCA), with the top 2000 highly variable genes (default settings) as input, was performed on each library individually. The results were then normalised with sctransform [29] using 30 principal components (PCs). The data from different samples were then integrated using Seurat [26] package v3.1.0. Uniform Manifold Approximation and Projection (UMAP) [30] was used for data visualisation. Populations of cells with similar transcriptomic profiles were clustered using the Leiden algorithm [31]. A total of 14 clusters were identified (Supplementary Figure S2), and annotated according to known marker genes for epithelial cells (*EPCAM*, *KRT18*), fibroblasts (*SPARC*, *COL3A1*), myeloid cells (*CD14, S100A8, CD68*), and lymphocytes (*PTPRC*, *CD3D*, *CD79A*). Differentially expressed genes (DEGs) were determined using Wilcoxon’s rank sum test with Bonferroni correction for multiple tests. ComplexHeatmap was used to generate heatmaps for gene expression visualisation [32]. The epithelial cells were further extracted, re-normalised, and re-integrated. Cell clustering and dimensionality reduction were performed as described above. Gene set enrichment analysis (GSEA) [33] was done using the fgsea package [34] with default parameters. Input for GSEA was ranked by average log2 fold change derived from the *findmarker* function, comparing each cluster and other cells with parameter logfc.threshold = 0, min.pct = 0, and min.diff = −Inf, in order to keep all genes as the input. Hallmark gene sets were used to assess biological process and state of gene expression [35]. In addition, publicly available data of normal gastrointestinal tract obtained from GSE125970 [36], were re-processed using the same pipeline as described above, and integrated with the single-cell data from this study.

## Results

### Collection of ascites from a CRC patient and cell preparation

**Figure 1:**
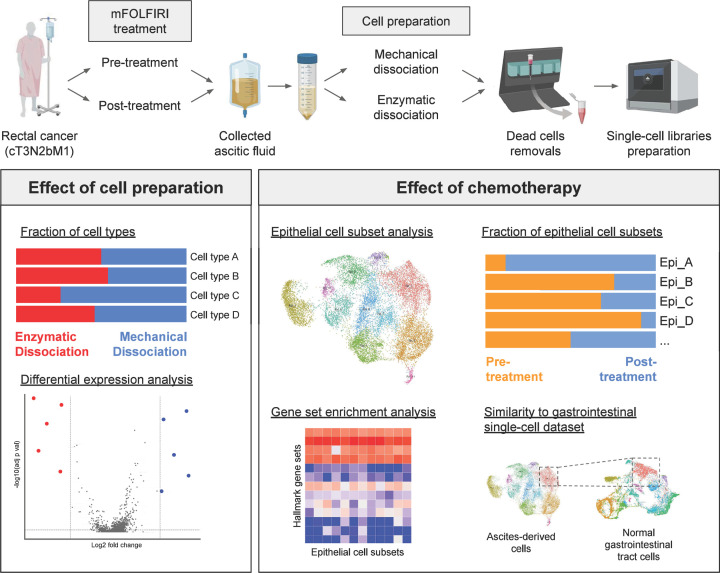
A summary flowchart showing the clinical course of the patient and study design Ascitic fluids were collected before and after a course of mFOLFIRI treatment in a patient with rectal cancer. The samples collected from each time point were prepared for single-cell transcriptomic analysis using either mechanical or enzymatic dissociation methods. All the samples were subjected to quality control and removal of dead cells, and only viable single-cell suspensions were used for the scRNA-seq experiment on the 10x Genomics platform (see also ‘Materials and methods’ section and Supplementary Figure S1 for more details). Subsequent data analysis explored the effect of sample preparation by comparing the fraction of captured cell types and DEGs between two preparation protocols. Further analyses investigated the effect of chemotherapy by comparing each fraction of epithelial cell subsets, GSEA, and comparing the expression profile to that of normal gastrointestinal scRNA-seq data.

Ascites-derived cells were collected from a CRC patient and processed as described in ‘Materials & methods’ section. Briefly, a 62-year-old woman had been diagnosed with advanced CRC (cT3N2bM1) with lung and liver metastases, and was under a course of first-line chemotherapy. However, the patient condition worsened due to intolerable side effects and she developed malignant ascites. Treatment regimen was then changed to mFOLFIRI. To investigate the treatment responsiveness of metastasised cancer cells, the ascites fluid samples, which were tapped and collected twice, before and after the first cycle of mFOLFIRI, were subjected to scRNA-seq profilings (see complete treatment scheme in Supplementary Figure S1). The samples were prepared by enzymatic and mechanical protocols, giving rise to a total of four samples to be further processed by scRNA-seq, namely Pre-tx enzymatic, Pre-tx mechanical, Post-tx enzymatic, and Post-tx mechanical. Accumax was selected as the enzyme of choice because it is less toxic and gentler on cells than trypsin and collagenase. Single-cell isolation, RNA extraction, and reverse transcription were carried out according to the 10x Genomics manufacturer’s protocols. Data analyses to elucidate the effect of different sample preparation protocols and the effect of chemotherapy on ascites cells were performed (Figure 1).

### Overall scRNA-seq profiles of pre- and post-chemotherapy CRC ascites-derived cells

**Figure 2:**
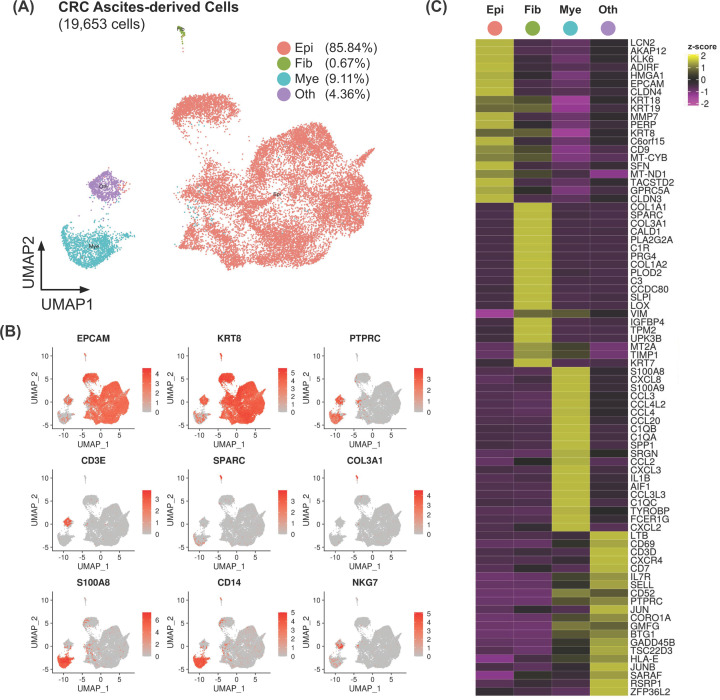
Profiling the pre- and post-chemotherapy CRC ascites-derived cells with scRNA-seq reveal heterogeneous cell populations across the samples (**A**) UMAP dimensional reduction plot of integrated data (four samples) overlaid with major cell type annotations. Epithelial cells accounted for the majority of the cells in the ascitic fluids from the patients. (**B**) UMAP dimensional reduction plot overlaid with normalised gene expression values of known marker genes of epithelial cells (*EPCAM*, *KRT8*), fibroblasts (*SPARC*, *COL3A1*), myeloid cells (*S100A8*, *CD14*), and other mixed lymphocytes (*PTPRC*, *CD3E*, *NKG7*). (**C**) Heatmap showing top ten marker genes for each of the major cell type (Epi, epithelial cells; Fib, fibroblasts; Mye, myeloid cells; Oth, other cells), as determined unbiasedly using Seurat *findmarker* function [26]. Yellow indicates relative overexpression as compared with other cell types, whereas purple indicates relative down-regulation.

From the four samples, we were able to profile transcriptional patterns of the total of 19,653 cells, with the number of cells from each individual sample ranging from 3,176 to 6,809 cells (Figure 2A, Supplementary Table S1). Interestingly, our scRNA-seq profiling revealed previously unappreciated heterogeneous cell populations comprising multiple cell types across the samples. In this particular case, the most abundant cell types found in the ascites-derived populations of cells were epithelial cells (85.84%), myeloid cells (9.11%), fibroblasts (0.67%), and other smaller populations of cells (4.36%). Figure 2B demonstrates the marker genes and their expression prevalence employed to identify the main populations (see also figure legend and ‘Materials and methods’ section for data clustering information and cell type classification). To further verify the cell types assigned, we used the function ‘FindMarker’ in the Seurat toolkits [26] to unbiasedly extract the most representative set of genes uniquely expressed in different populations, namely epithelial cells (*EPCAM, KRT8, KRT18*), fibroblasts (*SPARC, COL3A1, COL1A1*), and myeloid lineage (*S100A8, CXCL8, IL1B*) (Figure 2C). For ‘other’ smaller populations of cells, we observed the expression of *CD3E*, *CD79A* and *NKG7*, suggesting that this group of cells might contain a mixture of T cells, B cells, and NK cells.

### Choice of cell dissociation methods highly influenced scRNA-seq profiles

**Figure 3:**
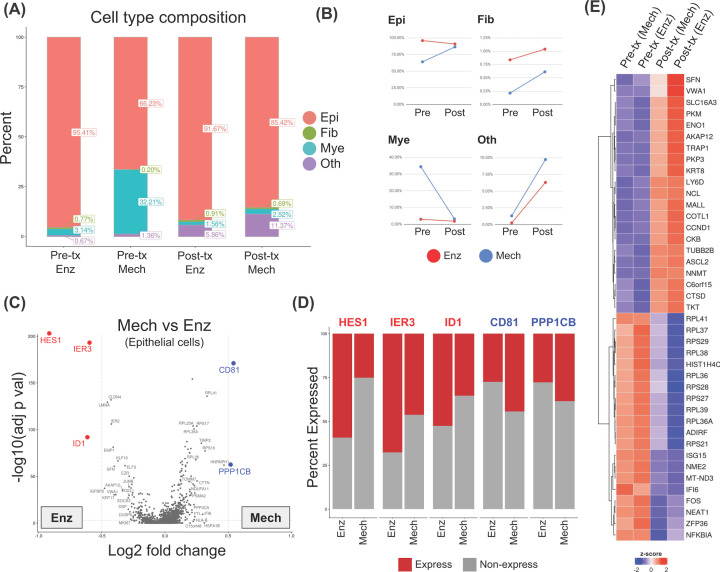
The effect of single-cell preparation methods (mechanical versus enzymatic dissociation) on gene expression (**A**) Bar graphs showing the fractions of the four major annotated cell types (Epi, epithelial cells; Fib, fibroblasts; Mye, myeloid cells; Oth, other cells) found in each sample and condition (Pre-tx, pre-treatment; Post-tx, post-treatment; Enz, enzymatic dissociation; Mech, mechanical dissociation). (**B**) Line plots showing the effects of sampling time point and preparation protocol on the frequency of each of the four major cell types. Dots and lines connect the pre- and post-treatment samples with the same preparation methods to show the trends between the two time points. (**C**) Volcano plots showing DEGs from the comparison between mechanical and enzymatic preparations of the epithelial cells, red and blue represent the up-regulated genes appeared in the enzymatic preparation as compared with mechanical preparation, and *vice versa* respectively. Coloured dots highlighted genes that have log2 fold change > 0.5 and adjusted *P*-value <0.01. (**D**) Bar plots showing a fraction of cells expressing DEGs from (C). Expressed fraction is determined by the number of cells having expression level more than quartile 1 (25%) of all the cells expressing that particular gene. (**E**) Heatmap showing DEGs from the comparison between the post- and pre-treatment epithelial cells. Red indicates relative overexpression as compared with other conditions, whereas blue indicates relative down-regulation.

We next asked if and how the methods of cell dissociation and/or that particular cycle of mFOLFIRI treatment had effects on the populations of identifiable cell types, as well as their gene expression profiles. Indeed, the most apparent differences in the percentages of recovered cell populations were between the enzymatic and mechanical dissociation protocols, especially between the two pre-treatment samples (Figure 3A,B). Strikingly, we observed that a higher proportion of myeloid cells were captured using the mechanical dissociation protocol (32.2 and 2.5%; Pre- and Post-tx), as compared with that of enzymatic preparation (3.1 and 1.6%; Pre- and Post-tx). As a result, the enzymatic preparation yielded slightly higher relative proportions of epithelial cells and fibroblasts, 95.4/91.7% and 0.8/0.9% respectively, as compared with 66.2/85.4% and 0.2/0.7% from the mechanical preparation samples.

In addition to the compositions of cell types found in the ascites samples, we also sought to determine whether the dissociation methods also affected the gene expression profiles. Differential expression (DE) analysis focusing on the epithelial cells obtained using the two dissociation methods showed that *HES1*, *IER3*, *JUNB*, *IER2, SOCS3*, and *ID1* were detected at significantly higher levels in the enzymatically dissociated samples than those obtained from the mechanically dissociated ones (Figure 3C,D and Supplementary Table S2). In addition, several genes encoding epithelial cell surface proteins and cytoskeletons such as *CLDN4* (Claudin 4), *SFN* (Stratifin), *LMNA* (Lamin A/C), *KRT17* (Keratin 17), and *EMP1* (Epithelial Membrane Protein 1) were also found at higher levels in the enzymatically dissociated samples. On the contrary, we found that *CD81* (Tetraspanin) and *PPP1CB* (Protein Phosphatase 1 Catalytic Subunit β) were under-represented in the enzymatically prepared samples. DE analyses of the myeloid cells showed that several chemokines and cytokine genes, such as *CCL3*, *CCL4*, *CXCL8*, *IL6*, and *IL1B* were found at low levels in the samples prepared by the enzymatic dissociation, in accordance with low percentages of myeloid cell population (Supplementary Figure S3). These results further demonstrate the effects of enzymatic and mechanical dissociations on not just the relative abundance of cell populations, but also on the gene expression profiles.

### Effect of the chemotherapy regimen, mFOLFIRI, on cellular heterogeneity and gene expression of ascitic cells

We observed that the proportions of the cells assigned to the epithelial cluster were largely unchanged before and after the treatment, as compared with the effect from the dissociation methods. Looking in more detail; however, the relative fractions of myeloid cells appeared to shrink slightly; whereas those of fibroblasts and the ‘others’ showed slightly increasing numbers in the samples from both dissociation methods (Figure 3A,B). We noted that, due to the limitation of the number of samples analysed here, these trends should be regarded as observations rather than confirmation, and thus would require further validation in additional patients.

Next, we sought to get an overview of transcriptomic profile changes of the epithelial cancer cells found in the ascites samples, before and after the mFOLFIRI treatment, by comparing the gene expression of epithelial cells as a whole, also known as ‘pseudo-bulk’ RNA-seq. DEGs are visualised using a heatmap (Figure 3E). After looking at genes that pass the selection threshold (log2 fold change > 0.5 and adjusted *P*-value <0.001), several ribosomal proteins encoded genes (e.g., *RPL36*, *RPL36A*, *RPL37*, *RPL38*, *RPL41*, *RPS21*, and *RPS29*) along with interferon-stimulated genes (*ISG15* and *IFI6*) are found to be lower expressed after the treatment. However, Gene Ontology (GO) term enrichment analyses of either up- or down-regulated genes did not result in any statistically significant gene set.

### scRNA-seq revealed treatment-susceptible and -resistant subpopulations

**Figure 4:**
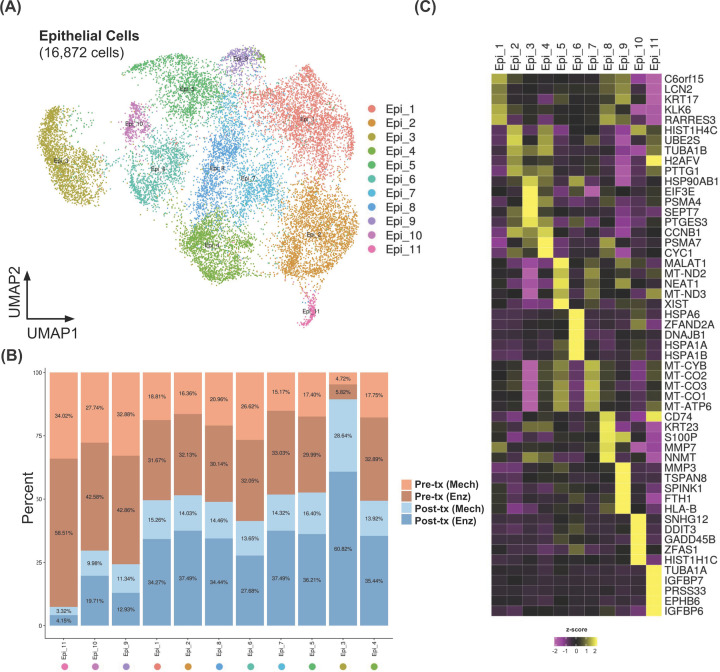
Epithelial cell clusters found in the CRC ascites were highly heterogeneous (**A**) UMAP of reclustered epithelial cells overlaid with subcluster annotations, showing gene expression heterogeneity even within the epithelial cells. (**B**) Bar plots showing sample composition of each subcluster in (A). (**C**) Heatmap showing the top five marker genes for each subcluster. Yellow indicates relative overexpression as compared with other subclusters, whereas purple indicates relative down-regulation.

As the pseudo-bulk analysis cannot fully demonstrate the changes of gene expression profiles of highly heterogeneous malignant ascitic cells, we therefore further analysed the epithelial cells in a greater depth by subsetting and re-clustering them based on their distinct transcriptomic profiles. In total, 11 transcriptionally distinct epithelial cell subclusters were annotated (Figure 4A,B). In the majority of the subclusters, the fractions of cells detected from different samples pre- and post-treatment, were largely comparable, except for the subclusters Epi_3, Epi_9, Epi_10, and Epi_11. The cells in Epi_3 were mainly from the post-treatment samples, regardless of the dissociation methods; whereas the cells in Epi_9, Epi_10, and Epi_11 were mainly found in the pre-treatment samples. This suggested possible differences in the degree of response to the treatment, as Epi_3 might be a relatively resistant population or clone that was able to expand after the treatment; whereas Epi_9, Epi_10, and Epi_11 might represent the clones that responded relatively well to that particular round of treatment.

As different epithelial cell subclusters possessed unique transcriptional characteristics, we next investigated the gene expression profiles of these subclusters, by obtaining the top five ‘marker genes’, or the most highly expressed genes in each cluster, as compared with the rest of the epithelial subclusters (Figure 4C). Among the diverse groups of marker genes identified, Epi_3 uniquely expressed a high level of genes encoding heat shock protein and proteasome (e.g., *HSP90AB1, PSMA4*). Epi_9’s marker genes include the members of matrix metalloproteinase and tetraspanin families (e.g., *MMP3, TSPAN8*), whereas Epi_10’s marker genes are related to DNA damage (e.g., *DDIT3, GADD45B*). Epi_11’s marker genes include the members of the insulin-like growth factor-binding protein family, IGFBP6 and IGFBP7, both of which are expressed in vascular endothelial cells and mesenchymal stromal cells [37–39]. The complete list of representative genes from each of the 11 clusters is shown in Supplementary Table S3.

### Possible biological mechanisms underlying the chemotherapy treatment susceptibility and resistance

**Figure 5:**
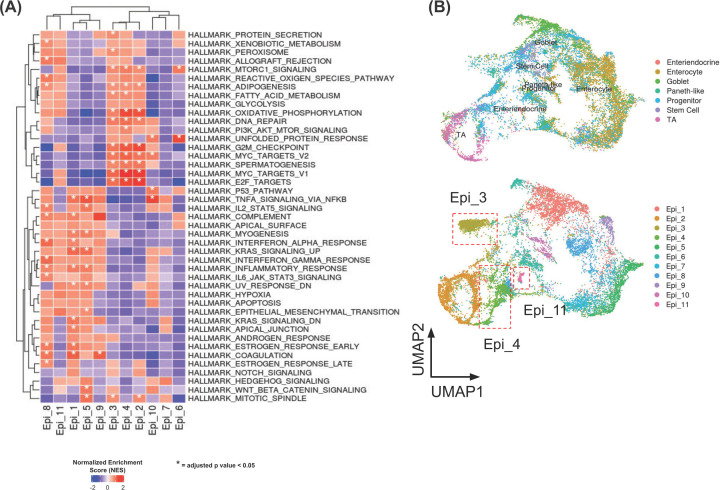
Functional gene set analysis of ascites-derived epithelial cells (**A**) Heatmap showing the normalised enrichment score from GSEA of hallmark gene sets from MSigDB [35] (*, adjusted *P*-value <0.05). Up-regulated genes in Epi_2, Epi_3, and Epi_4 were associated with hallmark gene sets in cell cycling, metabolism, and MTORC1 signaling pathways. (**B**) UMAP plot of integrated data between our malignant ascites single cell dataset and the normal gastrointestinal tract single cell dataset. Upper panel shows cells from normal gastrointestinal tract overlaid by original annotations. Lower panel shows epithelial subsets annotated as in Figure 4A. Epi_3 and Epi_4 showed slightest similarity when compared with normal gastrointestinal dataset.

We next investigated the putative functional profile of each subcluster based on the GSEA of the hallmark gene set collection from MSigDB [35] (Figure 5A). The signature genes of unfolded protein responses were highly represented in Epi_6, as several heat shock protein-coding genes including *HSPA6*, *HSPA1A*, and *HSPA1B*, were highly expressed in Epi_6. Whereas Epi_2, Epi_3, and Epi_4 were significantly enriched in the gene sets involved in cell cycling (mitotic spindle, G_2_/M checkpoint, E2F targets, MYC targets) and metabolism (oxidative phosphorylation, fatty acid metabolism). Only Epi_3 was uniquely enriched in protein secretion and peroxisome pathways. Epi_3, Epi_4, and Epi_6 were also enriched with the MTORC1 signaling pathway. The mammalian target of rapamycin (mTOR) is known to be involved in regulation of cell survival, tumour progression, and anti-cancer drug resistance in many types of cancer, including CRC [40]. Interestingly, the subclusters that appeared to respond to mFOLFIRI treatment, Epi_9, Epi_10, and Epi_11, did not show any statistically significant enrichment of the hallmark gene sets.

To further explore potential functions and biological relevances of these epithelial cell subclusters, we also compared our ascitic-derived scRNA-seq data from the CRC patient with the publicly available normal intestine scRNA-seq profiles [34] (Figure 5B). Among all the subclusters, we found that Epi_11 showed the most closely related expression profile to the normal enteroendocrine cells, of which are determined by close proximity coordination on the UMAP plot of integrated data. Epi_11 might potentially possess the most sensitive phenotype to the treatment, and thus it was almost completely eradicated from the post-treatment samples. Epi_9’s expression profile was closely related to normal enterocytes, whereas Epi_10’s expression profile is closely related to progenitor cells. The Epi_2 cluster showed close proximity to transit amplifying (TA) cells. TA cells are normally divided from normal stem cells and later differentiated into enterocytes [41,42]. Presence of gene expression profile of TA cells might reflect the stemness phenotype of cancer cells. Notably, the expression profiles of Epi_3 and Epi_4 only showed minimal similarity when compared with the public dataset; therefore, they appeared to represent the cell populations that were unique in malignant ascites samples. This suggested that Epi_3 and Epi_4 might be highly mutated cancer cells that did not share gene expression profiles with those of normal intestinal cells, as the other subclusters of ascites-derived cells did.

## Discussion

Single-cell transcriptomics has been used extensively to investigate several biological problems, cancer biology included, in the past decade [43]. Previous studies have investigated CRC at the single-cell resolution [22,44–48], and they have demonstrated the intratumoural heterogeneity and lineage development. In the present study, we have comprehensively investigated a case of advanced CRC using the ascites-derived cells, which can serve as a practical proxy for disease monitoring as it can be routinely collected from the patients undergoing abdominal paracentesis as part of the treatment. Through the gene expression analysis at single-cell resolution, we have showcased the intratumoural heterogeneity of cancer cells, the influence from cell preparation methods, and the changes of the cancer subpopulation landscape after a cycle of chemotherapy.

Ascites-derived cells have been used to study molecular mechanisms of cancers, including ovarian and gastrointestinal cancers, particularly to investigate disease progression and treatment responsiveness [49–55]. However, identification of biomarkers can be complicated by the heterogeneous cellular compositions. Using scRNA-seq profiling in conjunction with cell type identification based on characterised molecular markers, we were able to identify different cell types in ascites as well as their relative abundances. While epithelial cells were the most abundant populations (66–95% of all the cells retrieved from the ascites samples, depending on the cell dissociation methods, and the sample collection time points in relation to the chemotherapy treatment), we also observed fractions of myeloid cells (1–33%), fibroblasts (0.2–0.9%), as well as other subpopulations that were present at lower abundance.

One of the most striking findings of this work is the extent to which the cell preparation methods, enzymatic and mechanical cell dissociation, affected not just the relative proportions of cell types in the ascites samples, but also on the transcriptomic profiles of these subpopulations. As shown in muscle stem cells, van den Brink and co-workers found that a widely used cell preparation protocol [56], which involves tissue dissociation by collagenase type II followed by fluorescence-activated cell sorting (FACS), could significantly induce transcriptional changes. The ‘immediate early genes’ (IEGs) appeared to be specifically up-regulated in a subset of enzymatically treated cells, which might reflect the artifacts from the dissociation protocol. Other studies that compared the effects of different enzymatic dissociation methods also observed the expression of the same IEGs when performing dissociation at 37°C [57–59]. Consistent with these earlier studies, we observed highly represented genes in the ascites samples prepared using enzymatic dissociation, e.g., *HES1*, *IER3*, *JUNB*, *IER2*, *SOCS3*, and *ID1*, which had been previously identified by van den Brink co-workers [56] and O’Flanagan co-workers [58]. In addition, we observed that the myeloid cells in our samples were markedly susceptible to enzymatic dissociation by Accumax, which contains proteolytic and collagenolytic enzymes, especially in the pre-treatment samples. To the best of our knowledge, there is no previous report about the direct effect of enzymatic preparation on myeloid cells. Generally, this might be due to reduced cell viability after enzymatic treatment, plus cryopreservation.

Since our patient had been treated with mFOLFOX6 and CapeOx regimens before her ascites developed, this might have affected the viability of ascites-derived myeloid cell populations, resulting in more cell death after various manipulations. Moreover, as we observed the overall lower myeloid cell frequencies in the post-treatment ascites samples collected right after the first cycle of mFOLFIRI than in the pre-treatment samples regardless of preparation protocol, it is possible that this was the effect of cytotoxic chemotherapy-induced leukopenia. However, more careful investigations including further in-depth dissociation protocol comparisons for malignant ascites-derived cells are required to pinpoint the cause of this effect.

We compared the expression profiles of ascites-derived cells before and after a cycle of mFOLFIRI, comprising fluorouracil (5-FU), leucovorin, and irinotecan, which kill cancer cells via the inhibition of thymidylate synthase and topoisomerase I enzymes. However, due to the limitation of the sample size and sampling time points, it would be difficult to confidently investigate the specific impact of this chemotherapy regimen on the transcriptional changes and molecular pathways involved in the survival and progress of the cancerous cells. In spite of that, we have demonstrated the power of scRNA-seq in dissecting the heterogeneous subpopulations of metastasised cancer cells with distinct transcriptomic profiles. We have discovered that among the eleven epithelial subclusters, only three, namely Epi_9, Epi_10, Epi_11, seemed to be responsive to mFOLFIRI treatment, and one particular subcluster, Epi_3, could be considered a treatment-resistant population. This finding potentially reflects the poor outcome observed over this course of mFOLFIRI treatment in our patient.

We have also shown that these transcriptionally distinct cell populations also possessed unique functional characteristics, as the treatment-tolerant subpopulation, Epi_3, displayed the most divergent transcriptomic profile from that of any normal intestinal tissues. The cluster may represent a subclone with massive mutational events resulting in altered gene expression, which consequently allowed it to escape the chemotherapy treatment and became highly proliferated. Additionally, Epi_3 was uniquely enriched with genes in peroxisome pathways. Peroxisomes, which are reactive oxygen species (ROS)-degrading organelles, are known to play a role in therapeutic resistance in cancer when drugs inducing ROS-mediated apoptosis are involved [60], which is the case for both 5-FU and irinotecan [61,62]. On the contrary, the population of cells appeared to be the most susceptible to the treatment, Epi_9, Epi_10, and Epi_11, have relatively similar expression profiles as the normal enterocytes, progenitor cells, and normal enteroendocrine cells, respectively, which may explain why they are the most responsive to the cytotoxic treatment.

Taken together, we have provided one of the earliest studies where the groundbreaking scRNA-seq technology has been applied to explore the heterogeneity of the cells retrieved from malignant ascites. We have specifically demonstrated the cellular compositions of cell types found in the ascites samples, and showcased the under-appreciated impact of cell preparation protocols on the transcriptomic profiles of different cell types. Our results highlight the importance of using the optimised protocols in the scRNA-seq studies, and also emphasise the benefit of using scRNA-seq over the traditional bulk RNA-seq experiments, where the contributions to the overall expression from different cell types cannot be traced, in cancer research. Finally, we have provided an example of how scRNA-seq can be applied to routinely discarded ascites samples and resolve distinct subpopulations of cancer cells, in terms of both transcriptomic patterns, as well as cellular characteristics. Since malignant ascites is associated with advanced cancer and a poor prognosis, the potential usage of scRNA-seq to monitor real-time treatment response after chemotherapy initiation might help clinicians adjust or switch the regimens in a timely manner, which might extend the patients’ overall survival. Also, the collective interpretation of gene expression profiles of each subcluster should provide a more accurate prognosis for the cancer patients than the currently used bulk RNA-seq data. Further studies will be required to comprehensively validate the applications of scRNA-seq to discover new predictive and prognostic biomarkers from malignant ascites and other specimen types, as well as explore new molecular mechanisms and treatment options of complex diseases such as cancers.

## Supplementary Material

Supplementary Figures S1-S3 and Tables S1-S3

## Data Availability

scRNA-seq data discussed in this publication have been deposited in NCBI’s Gene Expression Omnibus and are accessible through the GEO Series accession number: GSE155953. All single-cell analyses and visualisations were performed in R version 3.6. Codes and scripts used in the bioinformatics analyses are available from the Github repository: https://github.com/vclabsysbio/scRNAseq_CAascites.

## Abbreviations

CapeOx: capecitabine/oxaliplatin
CRC: colorectal cancer
CT: computed tomography
DEG: differentially expressed gene
FOLFIRI: a chemotherapy regimen consisting of leucovorin calcium (calcium folinate), 5-fluorouracil, and irinotecan
FOLFOX: a chemotherapy regimen that include leucovorin calcium (calcium folinate), 5-fluorouracil and oxaliplatin
GSEA: gene set enrichment analysis
IEG: immediate early gene
mFOLFIRI: modified FOLFIRI
mFOLFOX6: modified FOLFOX6
PBMC: peripheral blood mononuclear cell
RBC: red blood cell
rcf: relative centrifugal force
scRNA-seq: single-cell RNA-sequencing
UMAP: Uniform Manifold Approximation and Projection
5-FU: fluorouracil
