# Supplementary material for: Capturing tumour heterogeneity in pre- and post-chemotherapy colorectal cancer ascites-derived cells using single-cell RNA-sequencing
Source: Biosci Rep. 2021 Dec 7;41(12):BSR20212093. doi: 10.1042/BSR20212093 (PMC8655500; doi:10.1042/BSR20212093)
Supplement: Supplementary Figures S1-S3 and Tables S1-S3 [file BSR-2021-2093_supp.pdf]

Supplementary Figure 1

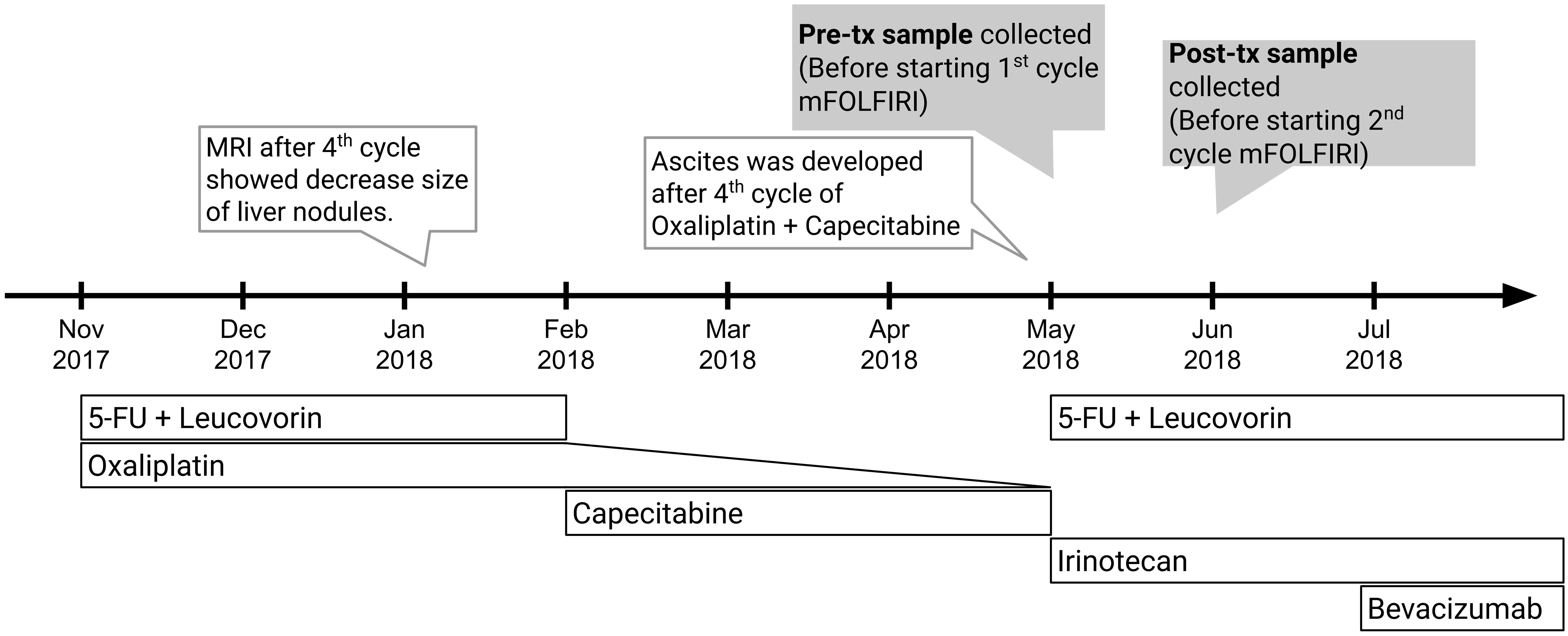

Supplementary Figure 1. A flowchart shows the complete timeline of treatment, clinical investigations, and clinical course of the patient in this study.

Supplementary Figure 2

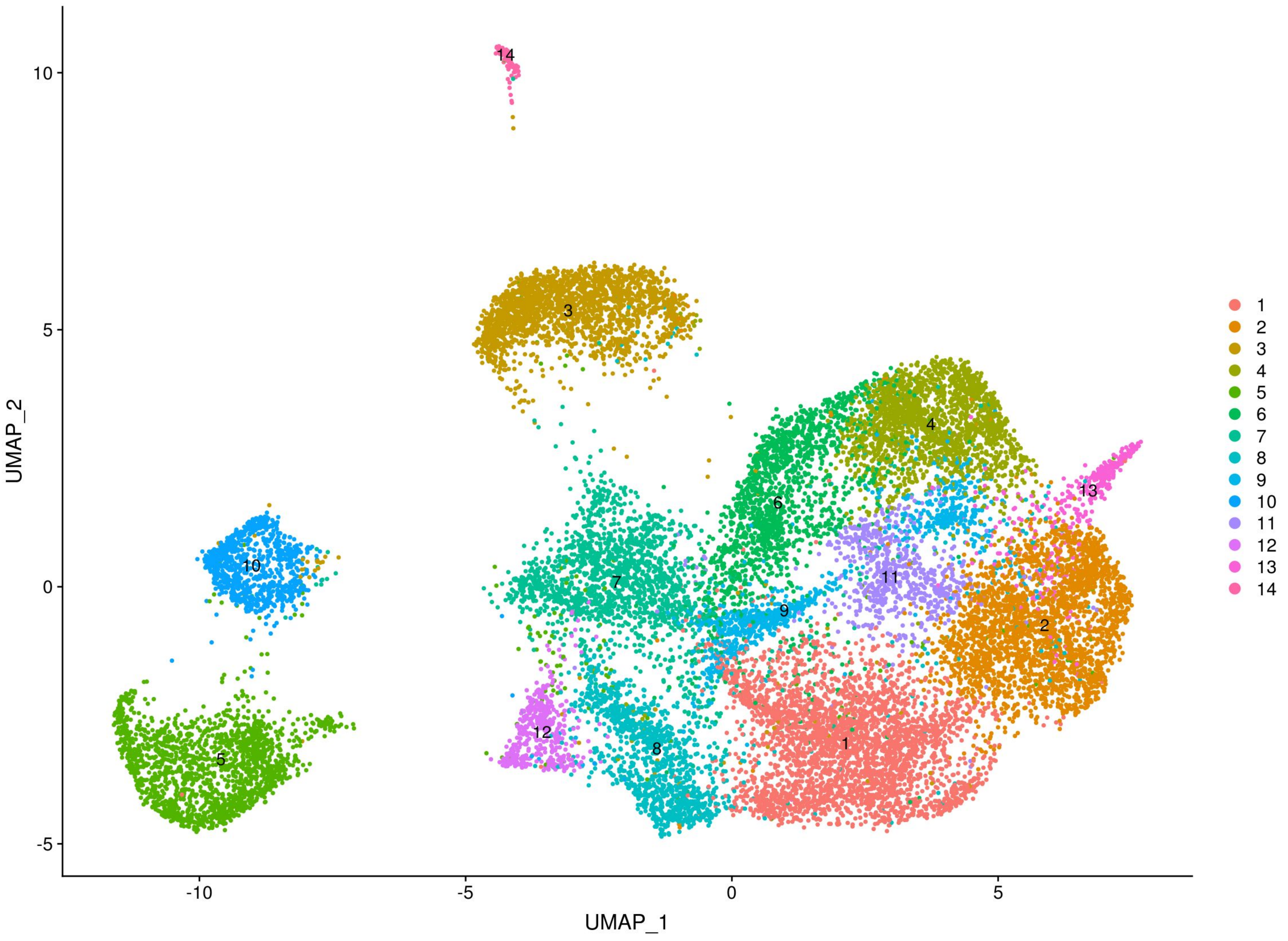

Supplementary Figure 2. UMAP plot showing the original Leiden clustering of the 14 sub-clusters of integrated data.

Supplementary Figure 3

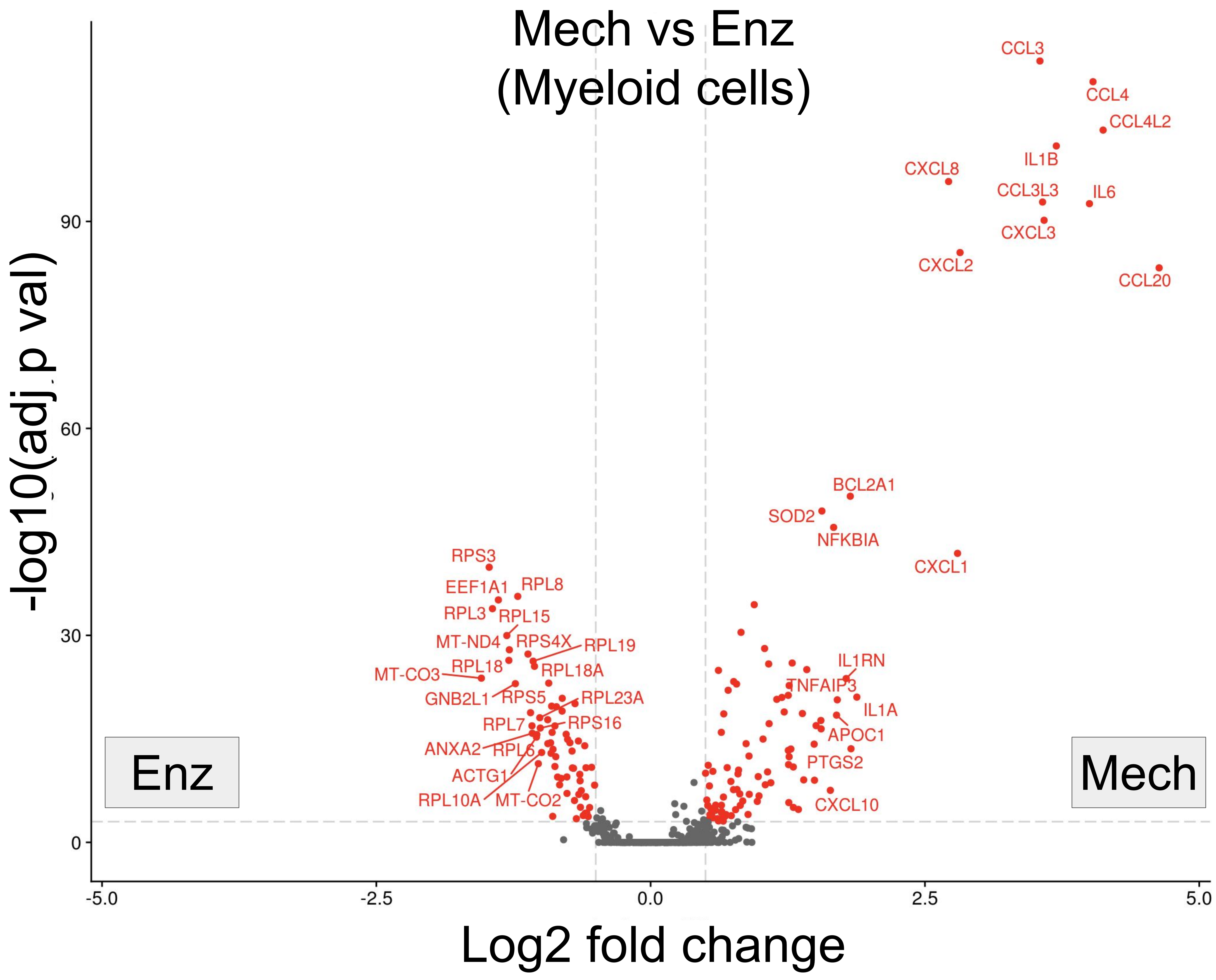

Supplementary Figure 3. Volcano plot showing DEGs from the comparison between mechanical and enzymatic preparations of myeloid cells. Colored dots highlighted genes that have log 2 fold change > 0.5 and adjusted p-value < 0.01.

Supplementary Table 1. Summary metric of scRNA-seq data output after read mapping and UMI quantification using Cellranger pipeline.

	CCM-B<br>Mech Pre-tx	CCM-A<br>Mech Post-tx	CCE-B<br>Enz Pre-tx	CCE-A<br>Enz Post-tx
Estimated Number of Cells	4,984	3,580	5,824	7,694
Mean Reads per Cell	41,940	62,594	37,882	28,570
Median Genes per Cell	1,685	3,076	2,528	2,388
Number of Reads	209,033,934	224,087,238	220,629,775	219,824,446
Valid Barcodes	97.80%	96.90%	97.90%	96.90%
Sequencing Saturation	60.00%	39.60%	55.80%	33.60%
Q30 Bases in Barcode	96.90%	97.30%	96.80%	97.30%
Q30 Bases in RNA Read	89.00%	90.90%	88.10%	90.80%
Q30 Bases in Sample Index	95.20%	95.30%	95.10%	95.50%
Q30 Bases in UMI	96.80%	97.20%	96.70%	97.20%
Reads Mapped to Genome	95.30%	97.20%	94.50%	97.30%
Reads Mapped Confidently to Genome	92.90%	95.10%	92.10%	95.20%
Reads Mapped Confidently to Intergenic Regions	3.20%	2.40%	2.90%	2.50%
Reads Mapped Confidently to Intronic Regions	11.90%	8.60%	11.80%	11.70%
Reads Mapped Confidently to Exonic Regions	77.80%	84.10%	77.40%	81.00%
Reads Mapped Confidently to Transcriptome	73.40%	79.40%	73.10%	76.60%
Reads Mapped Antisense to Gene	1.30%	1.20%	1.10%	1.30%
Fraction Reads in Cells	91.30%	76.00%	94.40%	87.90%
Total Genes Detected	21,833	21,766	21,713	21,929
Median UMI Counts per Cell	5,017	12,250	9,217	7,005

Supplementary Table 2. List of DEGs from the comparison between mechanical and enzymatic dissociations of epithelial cells (log2 fold change > 0.1).

gene	log2_avgFC	p_val_adj	pct.1	pct.2
CD81	0.5367	4.76E-171	0.57	0.377
PPP1CB	0.5152	1.14E-62	0.495	0.38
HNRNPH1	0.4667	8.75E-63	0.258	0.15
RPL41	0.3324	1.73E-136	0.913	0.885
RPS10	0.3172	6.84E-78	0.758	0.699
HSPA1B	0.3113	7.18E-01	0.412	0.372
IFI6	0.2986	3.67E-10	0.643	0.609
TIMP2	0.2881	3.15E-86	0.557	0.386
CTTN	0.2534	8.36E-46	0.512	0.377
RPS17	0.2514	2.14E-104	0.894	0.875
RPL26	0.2442	1.69E-68	0.903	0.885
FTL	0.2366	1.51E-07	0.932	0.947
PSMA2	0.2364	6.93E-33	0.587	0.474
NDUFA11	0.2335	1.30E-40	0.691	0.6
HLA-B	0.2314	1.98E-04	0.752	0.729
RPL35A	0.2297	1.59E-101	0.917	0.9
PPP3CA	0.2290	2.36E-20	0.262	0.191
TOMM7	0.2238	4.18E-42	0.752	0.698
C15orf48	0.2235	1.51E-02	0.468	0.431
RPL23A	0.2223	1.45E-104	0.934	0.931
RPS12	0.2170	6.25E-155	0.95	0.95
ZFAND2A	0.2134	5.66E-12	0.262	0.202
ZFAS1	0.2129	1.13E-14	0.736	0.69
NDUFA13	0.2097	8.70E-33	0.638	0.529
TRA2A	0.2070	7.64E-51	0.29	0.178
RPL21	0.2047	1.12E-30	0.899	0.879
RPL27	0.2045	1.75E-80	0.885	0.877
SNHG8	0.2021	6.14E-20	0.425	0.336
HSPA1A	0.1985	1.00E+00	0.336	0.308
COMMD6	0.1975	2.07E-34	0.717	0.641
IFI27	0.1961	4.18E-30	0.866	0.862
UQCR11	0.1960	1.18E-36	0.783	0.74
RPS25	0.1952	4.41E-76	0.926	0.918
RPS15A	0.1951	1.11E-75	0.92	0.902
FTH1	0.1949	1.07E-09	0.982	0.992
WTAP	0.1922	1.80E-20	0.289	0.211
COX7A2	0.1908	5.52E-45	0.814	0.794

SEC61G	0.1898	3.31E-21	0.768	0.737
FXYD3	0.1841	4.90E-12	0.659	0.598
UBA52	0.1817	1.14E-79	0.894	0.886
C6orf48	0.1790	3.40E-12	0.525	0.448
NDUFB2	0.1788	6.69E-25	0.77	0.743
RPL34	0.1760	2.48E-65	0.921	0.915
RARRES3	0.1737	3.27E-05	0.485	0.426
RPL28	0.1734	7.29E-88	0.943	0.94
ADIRF	0.1708	6.03E-23	0.856	0.851
SNHG7	0.1707	1.48E-12	0.525	0.445
RPL36A	0.1706	4.07E-29	0.761	0.663
OST4	0.1704	8.09E-32	0.787	0.765
SKP1	0.1667	6.52E-15	0.716	0.66
RPS13	0.1636	3.03E-61	0.899	0.897
RPS28	0.1629	1.66E-62	0.912	0.897
RPL35	0.1627	2.40E-56	0.921	0.915
MINOS1	0.1621	9.52E-20	0.651	0.585
S100A2	0.1620	4.75E-07	0.491	0.427
MRPL33	0.1613	3.04E-28	0.644	0.536
SERPINA1	0.1606	3.99E-04	0.329	0.282
B2M	0.1599	6.85E-04	0.91	0.912
RPLP2	0.1597	1.51E-82	0.96	0.959
TMEM59	0.1594	8.59E-17	0.604	0.509
RPL30	0.1587	9.40E-70	0.908	0.91
COX8A	0.1518	3.85E-23	0.757	0.731
TMED4	0.1509	3.31E-19	0.426	0.33
COX7C	0.1497	3.07E-26	0.833	0.817
RPL17	0.1486	2.86E-18	0.593	0.503
SAR1A	0.1485	4.02E-18	0.482	0.379
ITM2B	0.1484	8.09E-11	0.524	0.429
ARPC4	0.1481	1.35E-26	0.495	0.377
RPS27A	0.1471	5.43E-52	0.944	0.944
RPS21	0.1464	9.69E-28	0.834	0.816
POLR2L	0.1439	1.50E-13	0.76	0.73
MYEOV2	0.1430	2.18E-16	0.63	0.549
C4orf3	0.1421	5.08E-11	0.633	0.565
ATP5E	0.1399	2.60E-25	0.823	0.8
DNAJB1	0.1386	1.00E+00	0.564	0.526
UBL5	0.1381	1.54E-17	0.78	0.741
RPL27A	0.1378	2.69E-63	0.952	0.959
EIF2S3	0.1371	7.53E-30	0.496	0.376
BTG1	0.1354	1.00E+00	0.4	0.36
SMIM22	0.1354	7.06E-25	0.543	0.428
RPS19	0.1353	1.02E-66	0.968	0.974
LCN2	0.1347	1.00E+00	0.772	0.773
ATP5G2	0.1346	6.55E-13	0.725	0.683
RPLP1	0.1343	5.64E-51	0.962	0.97
BLOC1S1	0.1339	1.14E-19	0.541	0.443
TMEM256	0.1330	1.17E-30	0.482	0.36
RPS8	0.1330	8.06E-59	0.936	0.937
PARD6B	0.1322	2.45E-20	0.264	0.186

BLVRB	0.1314	7.25E-10	0.607	0.528
RPL32	0.1312	1.87E-56	0.953	0.96
ATP5J2	0.1311	2.45E-14	0.783	0.776
SNRPD2	0.1302	5.87E-11	0.751	0.711
RPL31	0.1293	8.88E-43	0.927	0.932
C12orf57	0.1292	7.08E-20	0.471	0.371
NDUFA1	0.1280	2.81E-16	0.723	0.651
RPS23	0.1280	1.14E-56	0.93	0.93
TSTD1	0.1278	2.62E-12	0.663	0.596
HERPUD1	0.1275	3.86E-06	0.287	0.234
HLA-A	0.1271	1.00E+00	0.814	0.805
RPL36	0.1263	1.22E-50	0.936	0.93
HPGD	0.1261	1.00E+00	0.653	0.636
RPS14	0.1238	2.30E-65	0.945	0.95
RPS26	0.1235	8.13E-25	0.816	0.808
RPS15	0.1235	9.39E-46	0.963	0.966
METRNL	0.1235	1.48E-20	0.262	0.192
RPS24	0.1234	3.34E-11	0.92	0.914
NDUFB4	0.1229	1.17E-12	0.743	0.713
RPS16	0.1220	4.74E-47	0.927	0.93
RPS20	0.1216	5.87E-38	0.907	0.905
ATOX1	0.1214	8.05E-14	0.575	0.489
IFI27L2	0.1212	2.51E-22	0.381	0.282
EIF5	0.1211	1.14E-10	0.609	0.52
RPL37A	0.1205	9.54E-32	0.868	0.863
NDUFA4	0.1187	1.08E-10	0.76	0.753
AXL	0.1185	2.21E-20	0.294	0.216
RPS18	0.1185	3.69E-55	0.974	0.982
MRPL52	0.1183	2.55E-17	0.595	0.499
CD47	0.1182	3.32E-13	0.399	0.317
IL18	0.1182	2.89E-03	0.654	0.605
MIA	0.1182	6.52E-21	0.405	0.308
TMSB10	0.1177	1.84E-20	0.961	0.968
RPL39	0.1175	1.92E-41	0.932	0.911
HINT1	0.1167	4.03E-12	0.799	0.78
TCEB2	0.1164	1.52E-18	0.809	0.801
RPL22	0.1162	7.08E-18	0.862	0.857
XBP1	0.1154	3.13E-04	0.351	0.291
RPL13A	0.1150	4.91E-28	0.952	0.961
RPS27L	0.1148	8.29E-12	0.574	0.488
CHCHD1	0.1142	1.23E-07	0.558	0.484
IAH1	0.1142	5.52E-11	0.551	0.462
MALAT1	0.1140	1.51E-03	0.878	0.915
SET	0.1140	9.37E-02	0.739	0.71
ZRANB2	0.1126	1.27E-22	0.392	0.286
HSPB1	0.1115	1.00E+00	0.855	0.855
LAMTOR2	0.1108	1.06E-15	0.569	0.47
MDK	0.1096	1.55E-06	0.468	0.4
PIN4	0.1094	2.85E-22	0.431	0.322
TCEAL4	0.1086	5.33E-22	0.438	0.33
BST2	0.1082	1.44E-03	0.486	0.431
RPS27	0.1081	2.62E-42	0.912	0.901

RNF181	0.1074	2.39E-08	0.489	0.41
NDUFB8	0.1073	2.38E-08	0.672	0.61
DBI	0.1067	3.73E-14	0.49	0.393
HSPA8	0.1059	1.54E-09	0.749	0.69
RPL12	0.1059	8.28E-18	0.939	0.947
PET100	0.1052	7.86E-22	0.461	0.343
RPL10A	0.1051	1.37E-22	0.892	0.889
TMEM258	0.1046	1.92E-13	0.764	0.73
SREK1IP1	0.1045	7.47E-20	0.333	0.24
VAMP8	0.1044	1.27E-06	0.535	0.466
FAU	0.1038	4.82E-36	0.907	0.913
KTN1	0.1037	4.03E-06	0.658	0.575
TAF1D	0.1034	1.15E-09	0.591	0.502
AIMP1	0.1033	2.50E-09	0.587	0.494
SERF2	0.1033	1.11E-11	0.848	0.857
S100A6	0.1032	2.78E-41	0.963	0.973
HBA1	0.1031	1.76E-12	0.329	0.258
ANAPC16	0.1031	1.09E-08	0.637	0.556
RP11-357H14.17	0.1031	2.09E-06	0.536	0.456
RABAC1	0.1030	2.98E-06	0.6	0.528
N4BP2L2	0.1018	7.91E-11	0.31	0.237
RPS5	0.1014	1.02E-18	0.903	0.908
PSME1	0.1008	4.60E-03	0.656	0.591
EIF3E	0.1007	5.09E-01	0.779	0.733
SND1	-0.1002	1.00E+00	0.315	0.312
IQGAP1	-0.1002	1.00E+00	0.363	0.32
TMEM123	-0.1003	2.17E-05	0.806	0.81
H1F0	-0.1006	1.00E+00	0.443	0.415
MAD2L1	-0.1007	1.00E+00	0.356	0.354
PARP1	-0.1008	1.00E+00	0.38	0.359
TSSC1	-0.1008	1.00E+00	0.385	0.356
TRIP6	-0.1011	1.00E+00	0.445	0.413
KLF6	-0.1016	1.07E-05	0.752	0.756
EWSR1	-0.1018	1.00E+00	0.42	0.393
PTGES2	-0.1021	1.00E+00	0.477	0.436
PSMD3	-0.1023	1.00E+00	0.539	0.496
VASP	-0.1027	1.00E+00	0.379	0.37
IFRD2	-0.1029	1.00E+00	0.437	0.405
IFT57	-0.1030	1.00E+00	0.267	0.248
BMP7	-0.1030	1.00E+00	0.324	0.298
AGPAT2	-0.1032	1.00E+00	0.59	0.544
CFL1	-0.1034	4.20E-11	0.83	0.847
BRD4	-0.1034	1.00E+00	0.377	0.351
MCM3	-0.1036	1.00E+00	0.281	0.242
SPAG7	-0.1037	2.46E-01	0.278	0.294
MYH9	-0.1037	1.00E+00	0.4	0.366
GNB1	-0.1037	1.00E+00	0.429	0.381
DNPEP	-0.1037	2.81E-03	0.329	0.343
TMEM11	-0.1039	1.00E+00	0.287	0.279
ASAP2	-0.1041	2.68E-01	0.355	0.355
MT-ND4	-0.1041	1.00E+00	0.791	0.847

ENTPD6	-0.1043	1.00E+00	0.324	0.309
RNF126	-0.1044	1.00E+00	0.483	0.449
CALU	-0.1044	1.00E+00	0.507	0.475
MRPS26	-0.1045	1.00E+00	0.541	0.501
PGM2L1	-0.1045	1.00E+00	0.285	0.274
TNFRSF1A	-0.1045	1.00E+00	0.308	0.308
RRP9	-0.1047	1.00E+00	0.297	0.285
PLOD1	-0.1048	1.00E+00	0.269	0.266
WIPI2	-0.1049	1.00E+00	0.363	0.339
RALY	-0.1051	3.65E-01	0.526	0.497
LBR	-0.1052	1.00E+00	0.313	0.317
CCT3	-0.1054	1.00E+00	0.64	0.595
SLMAP	-0.1056	1.00E+00	0.292	0.295
BRIX1	-0.1057	1.00E+00	0.348	0.328
PDIA4	-0.1061	1.00E+00	0.507	0.476
HNRNPR	-0.1062	1.00E+00	0.477	0.463
MRPS2	-0.1062	1.00E+00	0.434	0.414
SHISA5	-0.1065	1.00E+00	0.534	0.483
STMN1	-0.1066	5.90E-01	0.611	0.612
TBRG4	-0.1066	1.00E+00	0.323	0.305
UBE2C	-0.1067	1.00E+00	0.555	0.567
TMC6	-0.1069	1.00E+00	0.251	0.254
GNAS	-0.1071	1.00E+00	0.507	0.465
RCC1	-0.1075	1.00E+00	0.344	0.328
IER5	-0.1078	2.20E-07	0.284	0.31
SPAG9	-0.1085	1.00E+00	0.481	0.44
RSL1D1	-0.1085	6.97E-01	0.653	0.627
MAPRE1	-0.1086	1.00E+00	0.47	0.426
MVP	-0.1086	1.00E+00	0.38	0.377
SLC39A3	-0.1088	9.94E-03	0.318	0.332
CLTC	-0.1088	1.00E+00	0.357	0.323
TRIM28	-0.1089	1.00E+00	0.378	0.345
SLC38A5	-0.1089	1.00E+00	0.33	0.329
SUZ12	-0.1091	1.00E+00	0.369	0.365
SGK1	-0.1092	1.00E+00	0.301	0.304
GRB2	-0.1093	7.90E-01	0.361	0.356
ROCK2	-0.1094	1.00E+00	0.319	0.29
LLGL2	-0.1094	1.00E+00	0.513	0.462
CLPTM1	-0.1095	1.00E+00	0.32	0.312
SLC25A39	-0.1095	5.87E-02	0.701	0.678
PPAN	-0.1099	1.87E-04	0.292	0.316
TPM4	-0.1101	3.66E-01	0.681	0.66
RASSF7	-0.1106	3.07E-01	0.382	0.389
RAD23B	-0.1109	1.00E+00	0.424	0.382
GATAD2A	-0.1110	1.00E+00	0.333	0.288
SEPT9	-0.1112	1.00E+00	0.433	0.401
RPL7L1	-0.1112	1.00E+00	0.408	0.375
CDK12	-0.1113	1.00E+00	0.367	0.355
B3GAT3	-0.1115	1.00E+00	0.334	0.34
STAU1	-0.1118	1.24E-01	0.539	0.519
SNRPA	-0.1119	1.00E+00	0.329	0.31
RAB7A	-0.1119	6.79E-03	0.653	0.623

SEPT2	-0.1121	1.00E+00	0.292	0.295
YDJC	-0.1122	1.00E+00	0.556	0.527
RCN1	-0.1123	1.00E+00	0.689	0.648
OTUB1	-0.1126	1.00E+00	0.414	0.404
SSBP4	-0.1126	1.00E+00	0.399	0.396
WDR18	-0.1128	2.07E-03	0.392	0.398
LDHA	-0.1130	2.62E-03	0.84	0.857
FAM83H	-0.1132	1.00E+00	0.264	0.239
C1QBP	-0.1133	7.83E-01	0.654	0.64
HES6	-0.1133	1.00E+00	0.25	0.25
TPGS1	-0.1135	3.32E-03	0.295	0.316
H2AFX	-0.1135	7.82E-01	0.332	0.335
GAR1	-0.1136	1.00E+00	0.376	0.378
NUDT15	-0.1137	1.00E+00	0.268	0.273
TPX2	-0.1139	1.00E+00	0.309	0.299
RAD21	-0.1139	1.00E+00	0.544	0.507
SMC1A	-0.1144	1.00E+00	0.317	0.299
HDLBP	-0.1144	1.00E+00	0.411	0.396
XRCC6	-0.1147	1.00E+00	0.553	0.512
SCAMP4	-0.1147	3.46E-04	0.258	0.277
EGR1	-0.1147	4.39E-01	0.573	0.573
FUS	-0.1149	3.36E-06	0.609	0.59
SSRP1	-0.1152	1.00E+00	0.475	0.458
IGF2BP2	-0.1155	1.00E+00	0.424	0.381
TUBG1	-0.1157	1.00E+00	0.343	0.333
KLK6	-0.1158	4.26E-01	0.771	0.789
SURF4	-0.1158	3.85E-02	0.384	0.389
NOLC1	-0.1159	1.00E+00	0.472	0.425
EHF	-0.1160	1.00E+00	0.544	0.516
PLIN3	-0.1164	1.00E+00	0.533	0.486
GOT2	-0.1165	1.00E+00	0.291	0.291
GTPBP4	-0.1168	1.00E+00	0.401	0.384
STK25	-0.1173	1.46E-06	0.271	0.3
FKBP8	-0.1173	3.64E-02	0.651	0.613
HSP90B1	-0.1174	1.28E-03	0.768	0.769
MDFI	-0.1176	1.17E-01	0.261	0.271
TUBB	-0.1177	2.17E-02	0.701	0.693
SLC1A5	-0.1178	1.00E+00	0.354	0.351
LSR	-0.1180	1.88E-01	0.623	0.586
A4GALT	-0.1180	1.00E+00	0.374	0.329
RPUSD1	-0.1186	1.90E-02	0.28	0.294
CCT7	-0.1188	1.00E+00	0.532	0.484
CGREF1	-0.1189	1.00E+00	0.274	0.266
WDR43	-0.1189	1.00E+00	0.452	0.424
TUFM	-0.1191	3.23E-02	0.647	0.619
ACOT7	-0.1194	1.00E+00	0.472	0.437
PES1	-0.1196	1.00E+00	0.25	0.234
HNRNPU	-0.1198	1.00E+00	0.524	0.496
CSE1L	-0.1201	1.00E+00	0.355	0.343
TUBA1C	-0.1202	3.14E-02	0.641	0.631
PSMB1	-0.1205	1.44E-08	0.742	0.752
CTSV	-0.1206	1.00E+00	0.348	0.341

CDV3	-0.1209	1.76E-02	0.661	0.632
DBNL	-0.1212	9.78E-01	0.399	0.388
GALNT1	-0.1213	1.00E+00	0.307	0.311
LINC00657	-0.1223	1.00E+00	0.394	0.357
DIRAS3	-0.1224	1.00E+00	0.355	0.349
ACTB	-0.1225	9.43E-18	0.918	0.935
IL20RA	-0.1226	1.00E+00	0.554	0.535
SIRT7	-0.1228	3.46E-01	0.314	0.32
SERTAD1	-0.1232	1.00E+00	0.353	0.345
H2AFZ	-0.1234	6.21E-05	0.732	0.751
CAPN1	-0.1236	1.00E+00	0.58	0.544
PLK2	-0.1236	1.00E+00	0.402	0.385
MT-CYB	-0.1237	1.01E-02	0.758	0.818
CORO1C	-0.1240	1.00E+00	0.271	0.272
MFSD12	-0.1240	1.00E+00	0.314	0.309
IMPDH1	-0.1244	4.33E-05	0.289	0.313
WDR34	-0.1245	1.00E+00	0.453	0.418
TGFBI	-0.1248	1.00E+00	0.525	0.496
PPP1CA	-0.1248	6.58E-02	0.682	0.655
EPCAM	-0.1249	3.55E-10	0.804	0.827
GADD45A	-0.1249	3.84E-31	0.473	0.516
C8orf82	-0.1250	5.19E-06	0.283	0.311
CCNB1	-0.1251	1.00E+00	0.31	0.313
SRSF2	-0.1252	4.35E-04	0.643	0.617
KDELR2	-0.1255	1.17E-01	0.659	0.633
PPFIBP1	-0.1256	1.00E+00	0.427	0.417
EI24	-0.1259	1.00E+00	0.558	0.521
POLDIP2	-0.1273	1.00E+00	0.433	0.409
WBP11	-0.1274	1.00E+00	0.431	0.407
NR2F6	-0.1275	4.49E-01	0.487	0.46
TMEM259	-0.1275	1.00E+00	0.322	0.313
TUBB2A	-0.1278	1.00E+00	0.478	0.451
ARRDC1	-0.1281	1.00E+00	0.511	0.485
KHDRBS1	-0.1282	1.00E+00	0.504	0.475
STMN3	-0.1284	1.00E+00	0.426	0.348
MARCKSL1	-0.1286	3.46E-01	0.591	0.569
POR	-0.1287	1.00E+00	0.3	0.301
COL6A1	-0.1291	3.28E-02	0.25	0.268
HDGF	-0.1292	9.82E-02	0.509	0.477
HNRNPD	-0.1301	1.00E+00	0.584	0.557
DDB1	-0.1302	1.00E+00	0.305	0.288
RUVBL2	-0.1304	1.00E+00	0.482	0.447
JUP	-0.1308	1.00E+00	0.584	0.556
HBEGF	-0.1314	1.00E+00	0.29	0.291
NELFB	-0.1317	1.00E+00	0.283	0.279
CCT6A	-0.1318	1.27E-02	0.665	0.645
BCLAF1	-0.1325	5.77E-01	0.426	0.417
EPRS	-0.1325	3.07E-04	0.342	0.365
EPHA2	-0.1328	1.00E+00	0.364	0.34
LRRC59	-0.1329	5.52E-04	0.652	0.625
RTN4	-0.1333	7.02E-09	0.747	0.737
SDC1	-0.1334	5.60E-10	0.372	0.395

THRAP3	-0.1336	1.00E+00	0.467	0.434
SLC7A5	-0.1338	1.00E+00	0.275	0.274
TUBB6	-0.1340	1.00E+00	0.316	0.311
HMGN2	-0.1342	1.46E-05	0.645	0.664
ITGA3	-0.1345	1.00E+00	0.395	0.368
HGS	-0.1347	1.00E+00	0.434	0.405
PDLIM7	-0.1349	5.58E-02	0.367	0.368
ANKRD11	-0.1356	1.00E+00	0.437	0.423
HSF1	-0.1363	1.00E+00	0.474	0.438
PHLDB2	-0.1367	1.76E-01	0.341	0.355
CAV1	-0.1367	2.73E-05	0.717	0.716
EIF4A3	-0.1372	1.00E+00	0.468	0.44
RHBDD2	-0.1372	1.37E-01	0.295	0.304
CSNK1D	-0.1374	1.14E-01	0.306	0.314
CCDC86	-0.1377	9.94E-03	0.283	0.298
PRSS22	-0.1377	8.36E-05	0.562	0.581
UBC	-0.1382	7.30E-32	0.847	0.859
SPTBN1	-0.1386	1.00E+00	0.477	0.445
NCL	-0.1389	9.07E-07	0.696	0.702
LGALS3BP	-0.1393	1.84E-06	0.64	0.613
SEMA3B	-0.1394	1.00E+00	0.428	0.419
VPS51	-0.1402	2.77E-06	0.269	0.294
DDX39A	-0.1403	2.89E-02	0.325	0.335
SF1	-0.1403	7.90E-07	0.484	0.482
ALYREF	-0.1404	1.10E-05	0.27	0.294
MAD1L1	-0.1408	2.62E-07	0.266	0.299
CDC37	-0.1408	2.53E-03	0.618	0.583
RBM42	-0.1409	1.00E+00	0.452	0.417
EIF3I	-0.1413	1.82E-05	0.655	0.632
ZFP36L2	-0.1414	1.00E+00	0.446	0.426
CENPF	-0.1417	1.00E+00	0.281	0.3
TSC22D1	-0.1422	3.48E-06	0.644	0.64
DNAJC21	-0.1422	1.00E+00	0.306	0.303
MISP	-0.1423	6.16E-01	0.435	0.416
TOMM40	-0.1425	5.04E-01	0.515	0.489
CYP2W1	-0.1425	1.00E+00	0.294	0.294
ARF1	-0.1430	1.64E-13	0.709	0.69
RRP1	-0.1432	1.00E+00	0.32	0.312
VPS37B	-0.1433	4.55E-01	0.251	0.257
MCM7	-0.1435	1.00E+00	0.354	0.347
DST	-0.1441	1.00E+00	0.394	0.378
TK1	-0.1442	1.00E+00	0.452	0.45
KLF5	-0.1443	1.00E+00	0.301	0.305
CHPF	-0.1447	6.50E-01	0.389	0.388
BCL2L1	-0.1448	2.02E-06	0.583	0.581
SLC52A2	-0.1448	2.41E-04	0.465	0.453
DDX54	-0.1448	7.22E-04	0.302	0.313
MLF2	-0.1449	1.45E-06	0.651	0.632
BSG	-0.1451	2.11E-15	0.766	0.768
PSMD2	-0.1453	1.00E+00	0.486	0.461
EPS8L2	-0.1453	3.58E-02	0.412	0.405
LRRC61	-0.1454	1.00E+00	0.37	0.361

ST14	-0.1456	1.00E+00	0.496	0.46
RRBP1	-0.1456	1.00E+00	0.579	0.551
SPINT1	-0.1458	2.88E-01	0.545	0.52
SH3GLB2	-0.1461	1.14E-03	0.375	0.38
BPTF	-0.1464	7.49E-05	0.301	0.319
NOC2L	-0.1464	6.52E-01	0.337	0.332
HNRNPA0	-0.1467	1.41E-06	0.448	0.448
PDIA3	-0.1468	3.68E-02	0.685	0.647
DUSP1	-0.1469	1.30E-03	0.339	0.361
ITGA6	-0.1472	1.00E+00	0.352	0.353
VCP	-0.1472	1.00E+00	0.458	0.429
FAM84B	-0.1475	4.63E-03	0.279	0.293
AMN	-0.1487	1.65E-01	0.439	0.435
CCT5	-0.1498	1.00E+00	0.586	0.549
EFNB2	-0.1498	8.02E-03	0.31	0.318
RNH1	-0.1507	2.97E-03	0.636	0.596
YBX1	-0.1507	7.67E-24	0.875	0.895
LRRC8A	-0.1514	1.00E+00	0.31	0.294
THEM6	-0.1516	2.10E-04	0.399	0.401
PTPN12	-0.1517	1.00E+00	0.489	0.463
BAIAP2	-0.1520	1.00E+00	0.518	0.483
LIF	-0.1521	1.00E+00	0.257	0.269
MMP7	-0.1522	1.06E-09	0.696	0.728
SNX9	-0.1526	4.56E-05	0.34	0.357
SFPQ	-0.1529	1.00E+00	0.47	0.44
HPCAL1	-0.1534	9.08E-01	0.487	0.463
LY6D	-0.1535	1.21E-03	0.568	0.594
PUF60	-0.1538	1.00E+00	0.547	0.514
NAE1	-0.1541	4.30E-06	0.346	0.375
SAPCD2	-0.1544	1.28E-04	0.269	0.29
KRT18	-0.1546	1.42E-19	0.943	0.97
MROH6	-0.1547	2.16E-01	0.301	0.314
SNRPB	-0.1552	5.40E-07	0.695	0.697
PRKCDBP	-0.1553	1.00E+00	0.409	0.401
REPIN1	-0.1563	4.22E-06	0.306	0.323
EREG	-0.1565	1.63E-05	0.494	0.496
NES	-0.1567	1.00E+00	0.302	0.278
CDC42EP1	-0.1567	5.26E-04	0.502	0.489
PTRF	-0.1577	7.75E-03	0.381	0.385
TIMP3	-0.1577	1.00E+00	0.313	0.317
NCLN	-0.1580	4.04E-03	0.338	0.338
SMARCA4	-0.1583	4.66E-05	0.431	0.426
SYNCRIP	-0.1590	9.21E-03	0.591	0.571
UNC93B1	-0.1591	4.65E-04	0.35	0.355
SYNE2	-0.1593	1.00E+00	0.358	0.365
FOS	-0.1600	8.23E-06	0.64	0.64
SLC2A1	-0.1629	5.30E-10	0.7	0.693
GNG4	-0.1639	6.86E-04	0.467	0.459
EIF4H	-0.1647	4.71E-09	0.565	0.551
PVRL2	-0.1669	1.33E-06	0.376	0.387
LRRFIP1	-0.1672	5.12E-06	0.525	0.522
TM4SF1	-0.1677	5.93E-26	0.771	0.79

FBXW5	-0.1677	1.58E-05	0.385	0.391
SERPINB5	-0.1682	4.02E-04	0.294	0.317
KRT20	-0.1692	6.44E-04	0.659	0.661
TCEB3	-0.1694	5.19E-03	0.304	0.317
EHD1	-0.1706	1.78E-02	0.365	0.354
DUS1L	-0.1712	1.79E-05	0.454	0.441
TUBA1B	-0.1716	6.89E-06	0.684	0.693
ILF3	-0.1753	1.27E-01	0.468	0.448
PODXL2	-0.1754	1.43E-06	0.423	0.426
ANLN	-0.1767	4.57E-03	0.291	0.31
ARL4C	-0.1777	3.71E-05	0.38	0.39
CDC20	-0.1778	2.46E-03	0.339	0.361
PLEC	-0.1780	1.00E+00	0.501	0.456
DUSP5	-0.1790	3.49E-09	0.361	0.391
EIF4G1	-0.1798	5.79E-02	0.511	0.484
ACTG1	-0.1811	6.97E-32	0.891	0.914
P4HB	-0.1813	4.45E-18	0.738	0.73
EEF2	-0.1845	1.86E-22	0.816	0.831
PKN1	-0.1850	1.05E-11	0.327	0.355
PLAUR	-0.1853	3.69E-10	0.51	0.52
RAB8A	-0.1858	4.19E-07	0.353	0.362
ACTN4	-0.1858	1.41E-14	0.696	0.683
SLC20A1	-0.1873	1.00E+00	0.341	0.329
ETS2	-0.1875	3.30E-04	0.301	0.315
PKP3	-0.1907	1.46E-07	0.526	0.504
MGAT4B	-0.1907	4.72E-19	0.278	0.321
ADAP1	-0.1913	4.67E-15	0.419	0.451
ENO1	-0.1937	3.44E-11	0.776	0.777
ENC1	-0.1945	7.81E-12	0.267	0.308
MALL	-0.1947	1.06E-08	0.516	0.523
LAD1	-0.1952	2.92E-07	0.533	0.516
MT-CO3	-0.1958	8.99E-14	0.778	0.849
KRT8	-0.1965	9.60E-33	0.94	0.969
TINAGL1	-0.1983	2.09E-10	0.541	0.527
GIPC1	-0.2001	2.26E-06	0.59	0.555
RNF187	-0.2001	1.62E-11	0.298	0.321
URI1	-0.2007	1.90E-09	0.369	0.378
KLF3	-0.2013	4.70E-02	0.314	0.318
MT-ND4L	-0.2016	6.93E-09	0.564	0.583
UBALD2	-0.2020	8.97E-18	0.543	0.567
TRAP1	-0.2028	2.44E-05	0.429	0.421
PPP1R14B	-0.2036	1.29E-22	0.695	0.703
KRT23	-0.2047	1.00E+00	0.331	0.349
GJB3	-0.2066	7.26E-07	0.361	0.364
GPRC5A	-0.2079	1.17E-31	0.758	0.799
PTBP1	-0.2079	1.22E-17	0.427	0.449
SLC16A3	-0.2087	4.03E-09	0.642	0.617
UBE2S	-0.2094	3.72E-07	0.571	0.582
UBE2M	-0.2095	2.34E-11	0.437	0.442
COTL1	-0.2112	6.67E-13	0.509	0.518
PKM	-0.2126	5.67E-16	0.769	0.775
PIM3	-0.2182	2.68E-11	0.33	0.35

MIDN	-0.2189	1.51E-06	0.428	0.434
CCND1	-0.2192	1.87E-16	0.738	0.756
SOX9	-0.2284	1.30E-03	0.478	0.466
CKB	-0.2284	7.01E-13	0.629	0.645
BOP1	-0.2300	1.43E-12	0.455	0.46
CITED4	-0.2363	1.27E-09	0.394	0.393
EIF3B	-0.2367	5.07E-09	0.435	0.429
TRIB1	-0.2401	6.84E-17	0.302	0.344
EDN1	-0.2420	6.46E-03	0.393	0.404
ARHGDIA	-0.2449	1.44E-28	0.679	0.675
GDF15	-0.2464	1.24E-03	0.374	0.395
PHLDA2	-0.2469	4.96E-49	0.776	0.799
TUBB4B	-0.2475	5.87E-21	0.735	0.753
ZFP36	-0.2497	2.94E-12	0.424	0.458
LAMA3	-0.2511	3.90E-08	0.429	0.437
PLAU	-0.2516	1.87E-06	0.416	0.426
CLDN3	-0.2558	4.27E-33	0.729	0.763
RHOB	-0.2597	7.74E-31	0.713	0.726
ADM	-0.2624	1.23E-21	0.538	0.565
DUSP2	-0.2624	4.43E-42	0.251	0.331
IGFBP3	-0.2626	8.44E-04	0.5	0.494
LAMB3	-0.2639	3.32E-18	0.496	0.515
TACSTD2	-0.2694	2.91E-38	0.731	0.758
MKI67	-0.2720	6.32E-05	0.35	0.369
DUSP6	-0.2777	9.87E-13	0.535	0.534
EZR	-0.2834	7.04E-51	0.801	0.834
FOSL1	-0.2843	2.70E-30	0.472	0.514
JUNB	-0.2846	1.36E-39	0.688	0.704
DSP	-0.2905	1.74E-15	0.577	0.574
SOCS3	-0.2912	1.06E-27	0.362	0.404
ELF3	-0.3164	3.30E-62	0.724	0.756
AKAP12	-0.3350	1.57E-35	0.775	0.816
KLF10	-0.3721	3.10E-67	0.306	0.407
VWA1	-0.3886	3.62E-31	0.443	0.461
SFN	-0.4019	1.78E-61	0.719	0.763
KRT17	-0.4020	6.58E-31	0.565	0.603
EMP1	-0.4103	6.35E-82	0.661	0.699
IER2	-0.4245	8.67E-107	0.726	0.753
CLDN4	-0.4276	1.24E-132	0.777	0.839
LMNA	-0.4549	6.23E-130	0.741	0.786
IGFBP2	-0.4782	6.08E-38	0.326	0.378
IER3	-0.5982	3.70E-193	0.768	0.825
ID1	-0.6150	5.83E-92	0.573	0.649
HES1	-0.9168	0.00E+00	0.537	0.687

Supplementary Table 3. List of top 50 representative genes for each epithelial sub-cluster.

cluster	gene	log2_avgFC	p_val_adj	pct.1	pct.2
Epi_1	C6orf15	1.2641	0.00E+00	0.821	0.547
Epi_1	LCN2	1.2500	0.00E+00	0.983	0.727
Epi_1	KRT17	1.0443	0.00E+00	0.891	0.524
Epi_1	KLK6	0.9623	0.00E+00	0.974	0.741
Epi_1	PRSS22	0.8683	0.00E+00	0.847	0.515
Epi_1	TIMP3	0.8482	0.00E+00	0.59	0.256
Epi_1	KRT5	0.8249	0.00E+00	0.373	0.094
Epi_1	S100A4	0.8012	0.00E+00	0.966	0.811
Epi_1	CFD	0.7529	0.00E+00	0.841	0.533
Epi_1	BST2	0.7118	0.00E+00	0.773	0.378
Epi_1	KLK5	0.7010	0.00E+00	0.494	0.188
Epi_1	IL20RA	0.6846	0.00E+00	0.79	0.487
Epi_1	TMSB4X	0.6842	0.00E+00	0.999	0.958
Epi_1	RNASE1	0.6524	0.00E+00	0.447	0.141
Epi_1	GSN	0.6253	0.00E+00	0.8	0.478
Epi_1	C9orf16	0.6102	0.00E+00	0.969	0.739
Epi_1	KRT19	0.6032	0.00E+00	0.998	0.917
Epi_1	CD9	0.5853	0.00E+00	0.988	0.818
Epi_1	RABAC1	0.5833	0.00E+00	0.803	0.497
Epi_1	LEMD1	0.5755	0.00E+00	0.45	0.101
Epi_1	RPL3	0.5165	0.00E+00	0.995	0.918
Epi_1	EEF1A1	0.5128	0.00E+00	0.998	0.961
Epi_1	RPL10	0.5082	0.00E+00	0.998	0.952
Epi_1	CD24	0.6908	4.23E-299	0.909	0.638
Epi_1	CD63	0.5459	3.68E-298	0.962	0.741
Epi_1	S100A2	0.9454	4.96E-295	0.707	0.391
Epi_1	RARRES3	1.2046	3.66E-293	0.719	0.386
Epi_1	SERPINA1	0.9389	7.23E-289	0.554	0.241
Epi_1	GLIPR1	0.6308	6.17E-286	0.549	0.236
Epi_1	CST6	0.8692	4.22E-276	0.807	0.536
Epi_1	SAT1	0.7140	1.01E-275	0.964	0.745
Epi_1	TSPAN1	0.5907	2.44E-273	0.87	0.584
Epi_1	CYP2W1	0.7050	1.59E-252	0.537	0.241
Epi_1	SOX4	0.6315	4.95E-236	0.72	0.415
Epi_1	CLIC3	0.7429	1.24E-230	0.757	0.469
Epi_1	KLK7	0.5294	2.30E-227	0.735	0.431
Epi_1	PLA2G16	0.5130	9.17E-219	0.943	0.728
Epi_1	KLK1	0.5383	1.99E-217	0.402	0.157

cluster	gene	log2_avgFC	p_val_adj	pct.1	pct.2
Epi_1	RHOB	0.5039	6.27E-213	0.93	0.676
Epi_1	CTSV	0.5946	1.17E-203	0.558	0.296
Epi_1	PLAU	0.5713	5.85E-203	0.655	0.371
Epi_1	ID1	0.6527	5.45E-196	0.842	0.575
Epi_1	CTSD	0.5775	1.27E-188	0.896	0.691
Epi_1	CTSB	0.5100	7.92E-183	0.903	0.677
Epi_1	F3	0.5083	3.89E-154	0.771	0.519
Epi_1	AKAP12	0.6573	2.41E-145	0.922	0.776
Epi_1	ZFP36	0.5392	1.26E-136	0.635	0.405
Epi_1	MMP7	0.6442	2.93E-124	0.861	0.686
Epi_1	C15orf48	0.6516	6.25E-98	0.616	0.406
Epi_1	SPRR3	0.5891	4.01E-52	0.258	0.145
Epi_2	HIST1H4C	1.4444	0.00E+00	0.868	0.495
Epi_2	UBE2S	1.2225	0.00E+00	0.957	0.501
Epi_2	TUBA1B	1.0095	0.00E+00	0.982	0.63
Epi_2	H2AFV	0.9465	0.00E+00	0.929	0.44
Epi_2	PTTG1	0.9370	0.00E+00	0.877	0.379
Epi_2	H2AFZ	0.9234	0.00E+00	0.998	0.693
Epi_2	MKI67	0.9032	0.00E+00	0.81	0.271
Epi_2	RRM2	0.8931	0.00E+00	0.748	0.237
Epi_2	TUBB4B	0.8522	0.00E+00	0.971	0.701
Epi_2	CDKN3	0.8360	0.00E+00	0.807	0.289
Epi_2	RANBP1	0.8334	0.00E+00	0.98	0.626
Epi_2	DIRAS3	0.8321	0.00E+00	0.691	0.282
Epi_2	STMN1	0.7918	0.00E+00	0.948	0.542
Epi_2	HMGN2	0.7788	0.00E+00	0.984	0.59
Epi_2	CAV1	0.7708	0.00E+00	0.979	0.662
Epi_2	HMGB2	0.7680	0.00E+00	0.847	0.371
Epi_2	CCNB1	0.7601	0.00E+00	0.656	0.241
Epi_2	CENPW	0.7521	0.00E+00	0.881	0.378
Epi_2	SET	0.7346	0.00E+00	0.983	0.666
Epi_2	GAL	0.7126	0.00E+00	0.852	0.45
Epi_2	KIAA0101	0.7090	0.00E+00	0.7	0.232
Epi_2	SLC25A5	0.7078	0.00E+00	0.979	0.639
Epi_2	WDR34	0.6909	0.00E+00	0.781	0.357
Epi_2	TK1	0.6793	0.00E+00	0.83	0.372
Epi_2	PGAM1	0.6720	0.00E+00	0.937	0.571
Epi_2	DTYMK	0.6544	0.00E+00	0.748	0.301
Epi_2	NUCKS1	0.6503	0.00E+00	0.911	0.466
Epi_2	DUT	0.6329	0.00E+00	0.864	0.471
Epi_2	LDHB	0.6254	0.00E+00	0.991	0.712
Epi_2	PPP1R14B	0.6247	0.00E+00	0.957	0.648
Epi_2	ATP5G3	0.6183	0.00E+00	0.969	0.638
Epi_2	BIRC5	0.6113	0.00E+00	0.79	0.294
Epi_2	HMGB1	0.6068	0.00E+00	0.998	0.749
Epi_2	RAN	0.5988	0.00E+00	0.992	0.73
Epi_2	CCNB2	0.5913	0.00E+00	0.548	0.195
Epi_2	MZT2B	0.5880	0.00E+00	0.956	0.645
Epi_2	RHOBTB3	0.5843	0.00E+00	0.754	0.347
Epi_2	DNAJC9	0.5830	0.00E+00	0.763	0.341

cluster	gene	log2_avgFC	p_val_adj	pct.1	pct.2
Epi_2	CENPF	0.5770	0.00E+00	0.633	0.224
Epi_2	PHF19	0.5749	0.00E+00	0.592	0.184
Epi_2	CDC20	0.5748	0.00E+00	0.686	0.285
Epi_2	POLR2F	0.5695	0.00E+00	0.943	0.607
Epi_2	SNRPG	0.5617	0.00E+00	0.935	0.57
Epi_2	PRDX2	0.5586	0.00E+00	0.969	0.65
Epi_2	NDUFB2	0.5553	3.42E-302	0.982	0.705
Epi_2	TUBB	0.5947	5.10E-302	0.95	0.644
Epi_2	UBE2C	0.7721	7.22E-297	0.885	0.497
Epi_2	ENO1	0.5656	1.81E-290	0.989	0.733
Epi_2	IGFBP2	0.6022	2.44E-279	0.654	0.3
Epi_2	LGALS1	0.5795	4.01E-196	0.961	0.71
Epi_3	PTMA	0.5847	1.21E-189	0.931	0.961
Epi_3	HSP90AB1	0.7277	8.38E-103	0.745	0.91
Epi_3	HSP90AA1	0.6337	7.40E-94	0.77	0.899
Epi_3	RPL21	0.5809	9.40E-75	0.749	0.907
Epi_3	MORF4L1	0.4815	8.08E-45	0.272	0.588
Epi_3	PSMC5	0.4889	7.83E-44	0.287	0.618
Epi_3	SUMO1	0.5194	3.40E-42	0.284	0.605
Epi_3	HNRNPU	0.4822	4.74E-41	0.252	0.543
Epi_3	PSMA3	0.5022	4.83E-41	0.294	0.626
Epi_3	AIMP1	0.5468	4.93E-39	0.267	0.564
Epi_3	RAD21	0.5155	1.56E-35	0.272	0.556
Epi_3	HNRNPK	0.4889	1.70E-31	0.318	0.647
Epi_3	STIP1	0.4810	2.54E-31	0.271	0.543
Epi_3	SH3KBP1	0.6036	4.41E-30	0.271	0.544
Epi_3	CCT5	0.5364	5.37E-30	0.3	0.6
Epi_3	TPM3	0.5088	1.06E-29	0.337	0.679
Epi_3	GDI2	0.4938	2.39E-29	0.312	0.625
Epi_3	CALM2	0.5292	4.46E-29	0.659	0.899
Epi_3	ATP5A1	0.4959	1.85E-28	0.287	0.569
Epi_3	SRRM1	0.5137	6.18E-27	0.335	0.659
Epi_3	GLO1	0.6034	9.91E-27	0.301	0.594
Epi_3	CAPZA2	0.6529	2.78E-26	0.252	0.501
Epi_3	COTL1	0.5509	7.17E-25	0.286	0.549
Epi_3	BZW1	0.6663	1.51E-23	0.261	0.507
Epi_3	EIF5B	0.5616	3.46E-23	0.346	0.673
Epi_3	SNRPB2	0.5071	1.95E-22	0.33	0.634
Epi_3	LSM3	0.5470	7.67E-21	0.326	0.629
Epi_3	HMGB1	0.6781	2.67E-20	0.607	0.819
Epi_3	SSB	0.6696	8.19E-20	0.314	0.601
Epi_3	RSL24D1	0.6251	3.23E-17	0.322	0.607
Epi_3	PDAP1	0.5649	1.96E-16	0.357	0.676
Epi_3	CCT8	0.5924	1.20E-14	0.327	0.6
Epi_3	KPNA2	0.6106	1.57E-14	0.256	0.453
Epi_3	TMED2	0.6810	3.54E-14	0.335	0.616
Epi_3	HNRNPC	0.5210	4.99E-12	0.386	0.717
Epi_3	TAX1BP1	0.6379	2.57E-09	0.382	0.685
Epi_3	SNRPD2	0.5624	1.64E-08	0.414	0.77
Epi_3	EIF3E	0.9271	1.51E-07	0.527	0.782
Epi_3	TKT	0.5191	1.72E-07	0.412	0.738

cluster	gene	log2_avgFC	p_val_adj	pct.1	pct.2
Epi_3	NNMT	0.5931	4.39E-07	0.279	0.457
Epi_3	VDAC1	0.5162	4.32E-06	0.412	0.738
Epi_3	PSMA4	0.8497	2.42E-02	0.35	0.59
Epi_3	HMGB2	0.5902	1.00E+00	0.333	0.47
Epi_3	TMEM123	0.5288	1.00E+00	0.535	0.849
Epi_3	HMGN2	0.5458	1.00E+00	0.438	0.69
Epi_3	SEPT7	0.9121	1.00E+00	0.385	0.614
Epi_3	PTGES3	0.7229	1.00E+00	0.493	0.777
Epi_3	YWHAQ	0.5618	1.00E+00	0.464	0.755
Epi_3	UBE2C	0.5276	1.00E+00	0.417	0.585
Epi_3	NCL	0.7180	1.00E+00	0.461	0.735
Epi_4	TUBA1B	0.9153	0.00E+00	0.983	0.649
Epi_4	CCNB1	0.9062	0.00E+00	0.707	0.257
Epi_4	PSMA7	0.8744	0.00E+00	0.995	0.714
Epi_4	CYC1	0.8509	0.00E+00	0.981	0.622
Epi_4	C1QBP	0.8426	0.00E+00	0.96	0.602
Epi_4	SNRPB	0.8245	0.00E+00	0.986	0.656
Epi_4	EIF5A	0.7749	0.00E+00	0.984	0.642
Epi_4	LYZ	0.7597	0.00E+00	0.872	0.431
Epi_4	FABP5	0.7566	0.00E+00	0.965	0.571
Epi_4	PCNA	0.7292	0.00E+00	0.767	0.291
Epi_4	CTNNAL1	0.7189	0.00E+00	0.867	0.405
Epi_4	HMGB2	0.7100	0.00E+00	0.883	0.393
Epi_4	MAL2	0.7030	0.00E+00	0.979	0.642
Epi_4	ATP5A1	0.7010	0.00E+00	0.903	0.481
Epi_4	CDC20	0.6980	0.00E+00	0.747	0.299
Epi_4	AURKA	0.6927	0.00E+00	0.561	0.143
Epi_4	MRPS26	0.6830	0.00E+00	0.88	0.464
Epi_4	BIRC5	0.6732	0.00E+00	0.826	0.317
Epi_4	COPRS	0.6730	0.00E+00	0.892	0.48
Epi_4	FKBP1A	0.6687	0.00E+00	0.965	0.625
Epi_4	ATP5G1	0.6631	0.00E+00	0.951	0.58
Epi_4	PFN1	0.6590	0.00E+00	0.996	0.766
Epi_4	NME1	0.6438	0.00E+00	0.993	0.703
Epi_4	TMEM14B	0.6305	0.00E+00	0.911	0.509
Epi_4	TXNL4A	0.6258	0.00E+00	0.878	0.453
Epi_4	GCSH	0.6239	0.00E+00	0.813	0.364
Epi_4	CACYBP	0.6149	0.00E+00	0.93	0.527
Epi_4	COX5A	0.5930	0.00E+00	0.978	0.654
Epi_4	NDUFB9	0.5850	1.43E-303	0.994	0.718
Epi_4	UBE2S	0.7675	1.44E-301	0.945	0.528
Epi_4	LAPTM4B	0.5780	5.14E-300	0.789	0.376
Epi_4	YWHAQ	0.6157	6.10E-300	0.981	0.681
Epi_4	PA2G4	0.5971	4.90E-299	0.972	0.621
Epi_4	KPNA2	0.6326	6.30E-297	0.81	0.375
Epi_4	PSMB6	0.6038	6.99E-297	0.937	0.583
Epi_4	RAN	0.6076	2.35E-296	0.995	0.745
Epi_4	CKS1B	0.6180	2.58E-294	0.914	0.484
Epi_4	CKS2	0.6872	1.29E-291	0.852	0.425
Epi_4	DNPH1	0.5951	1.76E-276	0.927	0.553
Epi_4	H2AFZ	0.6560	2.15E-274	0.995	0.71

cluster	gene	log2_avgFC	p_val_adj	pct.1	pct.2
Epi_4	HMGN2	0.6253	9.06E-262	0.972	0.614
Epi_4	LDHB	0.5961	6.68E-250	0.994	0.727
Epi_4	PTTG1	0.5904	2.49E-248	0.855	0.41
Epi_4	MT1E	0.6558	1.76E-237	0.886	0.484
Epi_4	HIST1H4C	0.9081	8.88E-227	0.881	0.514
Epi_4	ENO1	0.6012	4.10E-219	0.993	0.747
Epi_4	UBE2C	0.8353	4.00E-217	0.883	0.519
Epi_4	MT2A	0.7232	1.73E-212	0.845	0.475
Epi_4	SLIRP	0.6271	8.94E-198	0.962	0.63
Epi_4	CKB	0.6556	1.02E-178	0.926	0.6
Epi_5	MALAT1	2.9357	0.00E+00	0.968	0.895
Epi_5	MT-ND2	1.7027	0.00E+00	0.903	0.846
Epi_5	MT-CO1	1.1799	1.44E-224	0.874	0.839
Epi_5	NEAT1	2.6064	1.09E-221	0.749	0.687
Epi_5	MT-ATP6	1.2772	2.43E-217	0.819	0.795
Epi_5	MT-ND1	1.2042	4.64E-212	0.846	0.842
Epi_5	MT-ND4	1.1277	1.55E-182	0.839	0.827
Epi_5	MT-ND5	1.3077	1.04E-147	0.747	0.791
Epi_5	MT-ND3	1.6868	9.52E-147	0.754	0.75
Epi_5	MT-CO3	1.0338	1.42E-117	0.835	0.823
Epi_5	MT-CYB	0.9495	7.07E-108	0.792	0.799
Epi_5	MT-CO2	0.8451	3.58E-104	0.83	0.804
Epi_5	XIST	1.7293	1.62E-96	0.463	0.331
Epi_5	MT-ND4L	1.3707	1.13E-54	0.554	0.579
Epi_5	SAT1	1.1190	1.64E-27	0.65	0.8
Epi_5	CCNL1	1.3398	3.32E-26	0.486	0.534
Epi_5	ELF3	1.0475	7.64E-26	0.598	0.763
Epi_5	GPRC5A	0.7711	5.10E-16	0.616	0.806
Epi_5	WSB1	1.0866	2.54E-14	0.274	0.228
Epi_5	SLC20A1	1.1907	4.11E-13	0.345	0.332
Epi_5	PLEC	1.1054	1.28E-11	0.419	0.478
Epi_5	N4BP2L2	1.0857	8.23E-10	0.284	0.258
Epi_5	ZNF292	1.0195	1.75E-08	0.27	0.243
Epi_5	JUND	1.1958	7.62E-08	0.362	0.382
Epi_5	KLF6	0.8623	8.24E-08	0.589	0.774
Epi_5	DST	0.9934	6.13E-07	0.36	0.386
Epi_5	LAMB3	1.0482	8.89E-07	0.432	0.518
Epi_5	LIF	0.9487	5.44E-06	0.279	0.263
Epi_5	LIPH	1.0827	1.47E-05	0.327	0.333
Epi_5	VMP1	1.2966	7.24E-05	0.41	0.496
Epi_5	POLR2J3	1.0337	1.36E-04	0.332	0.357
Epi_5	MIDN	1.0322	4.11E-04	0.383	0.438
Epi_5	SEMA3B	0.9849	1.08E-02	0.371	0.428
Epi_5	IFRD1	0.9275	1.23E-02	0.298	0.309
Epi_5	SLC38A2	0.9601	6.22E-02	0.32	0.35
Epi_5	PADI1	0.8720	9.18E-01	0.265	0.276
Epi_5	LAMA3	0.9011	1.00E+00	0.361	0.443
Epi_5	AKAP9	0.9115	1.00E+00	0.298	0.339
Epi_5	LMO7	0.8523	1.00E+00	0.345	0.41
Epi_5	SF1	0.9011	1.00E+00	0.382	0.494
Epi_5	SLC25A37	0.8320	1.00E+00	0.26	0.293

cluster	gene	log2_avgFC	p_val_adj	pct.1	pct.2
Epi_5	FUS	0.8543	1.00E+00	0.436	0.615
Epi_5	NPEPPS	0.8834	1.00E+00	0.313	0.384
Epi_5	PNISR	0.7934	1.00E+00	0.321	0.394
Epi_5	JUN	1.0376	1.00E+00	0.438	0.604
Epi_5	MACF1	0.7709	1.00E+00	0.295	0.371
Epi_5	FOSB	1.0348	1.00E+00	0.304	0.386
Epi_5	RNF213	0.8072	1.00E+00	0.292	0.364
Epi_5	ANKRD11	0.7997	1.00E+00	0.325	0.44
Epi_5	NAMPT	0.8524	1.00E+00	0.375	0.514
Epi_6	HSPA6	3.1143	0.00E+00	0.384	0.046
Epi_6	ZFAND2A	1.9023	0.00E+00	0.635	0.187
Epi_6	HSPB1	1.3175	9.52E-207	0.952	0.847
Epi_6	HERPUD1	1.0201	3.38E-203	0.559	0.225
Epi_6	DNAJB1	2.0658	1.44E-199	0.773	0.518
Epi_6	FTL	0.8417	7.76E-188	0.977	0.939
Epi_6	DNAJB9	0.6720	1.17E-184	0.401	0.123
Epi_6	BAG3	1.3564	2.76E-173	0.489	0.192
Epi_6	HSPA5	1.3696	7.15E-171	0.789	0.576
Epi_6	EIF1	0.5724	3.57E-170	0.965	0.895
Epi_6	HSPH1	1.4453	3.32E-162	0.671	0.388
Epi_6	HSPA1A	2.6801	1.34E-154	0.562	0.297
Epi_6	ZFAS1	0.9686	8.82E-154	0.882	0.691
Epi_6	SERP1	0.8338	2.40E-148	0.811	0.676
Epi_6	CRYAB	1.1485	6.38E-146	0.357	0.113
Epi_6	OSER1	0.7943	5.21E-123	0.523	0.258
Epi_6	DDIT3	0.6734	3.58E-116	0.39	0.146
Epi_6	OAZ1	0.6434	2.23E-113	0.937	0.824
Epi_6	SDF2L1	1.0734	1.40E-112	0.69	0.514
Epi_6	C6orf48	0.9322	1.42E-112	0.694	0.455
Epi_6	DNAJC3	0.5480	3.52E-112	0.459	0.206
Epi_6	HSPA1B	2.7510	1.37E-110	0.57	0.37
Epi_6	DEDD2	0.7856	1.94E-104	0.476	0.235
Epi_6	AGR2	0.7003	5.92E-103	0.333	0.127
Epi_6	SELK	0.7634	2.05E-100	0.675	0.485
Epi_6	VIMP	0.6587	5.56E-100	0.686	0.485
Epi_6	SERPINH1	1.1626	1.48E-99	0.541	0.314
Epi_6	CDK2AP2	0.6454	5.68E-84	0.575	0.379
Epi_6	SNHG7	0.7708	1.73E-83	0.654	0.456
Epi_6	TAF1D	0.6643	5.23E-83	0.709	0.517
Epi_6	EIF4A2	0.5590	3.91E-81	0.806	0.652
Epi_6	DNAJB11	0.5866	9.86E-76	0.553	0.368
Epi_6	DNAJA4	0.5699	2.62E-75	0.283	0.112
Epi_6	FKBP4	0.8433	3.41E-75	0.743	0.591
Epi_6	TAF7	0.8328	6.91E-71	0.624	0.429
Epi_6	PPP1R15A	0.9352	4.99E-67	0.707	0.519
Epi_6	KRT10	0.6335	2.33E-63	0.768	0.638
Epi_6	SAR1A	0.5848	2.36E-62	0.575	0.399
Epi_6	MANF	0.8589	6.60E-62	0.663	0.559
Epi_6	CACYBP	0.7197	5.02E-57	0.705	0.565
Epi_6	MT1X	0.7263	3.24E-56	0.347	0.176
Epi_6	MRPL18	0.8185	8.14E-50	0.67	0.547

cluster	gene	log2_avgFC	p_val_adj	pct.1	pct.2
Epi_6	YTHDF2	0.5722	2.94E-48	0.534	0.371
Epi_6	DNAJA1	0.7744	7.07E-36	0.577	0.467
Epi_6	PDIA4	0.5854	1.26E-31	0.564	0.48
Epi_6	HSPA8	0.6141	4.69E-27	0.803	0.702
Epi_6	STIP1	0.6534	7.06E-27	0.606	0.5
Epi_6	NUDC	0.6037	5.45E-24	0.764	0.689
Epi_6	SNRPB2	0.5517	1.42E-19	0.683	0.587
Epi_6	UBB	0.7628	9.20E-16	0.856	0.807
Epi_7	MT-CYB	1.4423	0.00E+00	0.994	0.782
Epi_7	MT-CO2	1.4228	0.00E+00	0.995	0.791
Epi_7	MT-CO3	1.3899	0.00E+00	0.998	0.81
Epi_7	MT-CO1	1.3789	0.00E+00	0.996	0.83
Epi_7	MT-ATP6	1.3520	0.00E+00	0.996	0.781
Epi_7	MT-ND4	1.3413	0.00E+00	0.998	0.814
Epi_7	MT-ND5	1.2718	0.00E+00	0.986	0.769
Epi_7	MT-ND1	1.2107	0.00E+00	0.999	0.829
Epi_7	MT-ND2	1.1164	0.00E+00	0.999	0.84
Epi_7	MT-ND3	1.0046	2.66E-186	0.978	0.732
Epi_7	MT-ND4L	0.8060	7.86E-167	0.813	0.557
Epi_7	CSTB	0.8011	3.28E-133	0.988	0.897
Epi_7	S100A11	0.5107	2.10E-128	0.998	0.948
Epi_7	ELF3	0.6695	7.59E-110	0.891	0.733
Epi_7	NEAT1	0.7480	2.91E-104	0.882	0.678
Epi_7	MALAT1	0.4484	1.33E-102	0.988	0.895
Epi_7	RPLP2	0.3748	3.44E-89	0.995	0.956
Epi_7	COX6B1	0.4539	2.45E-86	0.909	0.793
Epi_7	COX5B	0.3940	1.60E-81	0.946	0.826
Epi_7	S100A6	0.4450	7.99E-79	0.997	0.967
Epi_7	ADIRF	0.6323	3.15E-73	0.965	0.843
Epi_7	GPRC5A	0.5838	5.83E-69	0.912	0.775
Epi_7	TMA7	0.4979	2.76E-63	0.895	0.796
Epi_7	TXN	0.4193	5.79E-63	0.958	0.831
Epi_7	POLR2J3	0.4354	2.04E-62	0.522	0.341
Epi_7	CST3	0.4259	3.37E-62	0.928	0.817
Epi_7	FAM83H	0.4152	9.79E-62	0.417	0.234
Epi_7	PLEC	0.5080	3.08E-60	0.617	0.459
Epi_7	RRBP1	0.5775	2.11E-59	0.674	0.551
Epi_7	TMSB10	0.3839	9.12E-58	0.997	0.963
Epi_7	RPL37	0.4858	6.60E-55	0.981	0.88
Epi_7	RPL38	0.4692	8.73E-53	0.975	0.873
Epi_7	PDCD5	0.4247	1.18E-50	0.758	0.695
Epi_7	SHFM1	0.3857	2.60E-48	0.834	0.75
Epi_7	COX17	0.4297	4.99E-48	0.747	0.66
Epi_7	RND3	0.5611	5.24E-46	0.503	0.349
Epi_7	FAM25A	0.5336	1.54E-43	0.32	0.171
Epi_7	JUND	0.3970	1.33E-41	0.52	0.368
Epi_7	RPS21	0.3917	1.78E-40	0.918	0.814
Epi_7	RNF213	0.4501	4.32E-37	0.48	0.346
Epi_7	CDV3	0.4080	1.13E-35	0.704	0.637
Epi_7	MIDN	0.4379	3.19E-28	0.533	0.423
Epi_7	LY6D	0.5030	4.30E-28	0.691	0.576

cluster	gene	log2_avgFC	p_val_adj	pct.1	pct.2
Epi_7	JUN	0.3823	4.48E-28	0.671	0.579
Epi_7	FOSB	0.3944	3.24E-21	0.473	0.369
Epi_7	MUC1	0.3782	4.03E-20	0.413	0.315
Epi_7	MT2A	0.5162	1.35E-19	0.612	0.512
Epi_7	UPK2	0.4495	1.75E-15	0.529	0.447
Epi_7	LAMA3	0.4057	5.95E-15	0.492	0.429
Epi_7	SLC2A3	0.4928	1.17E-12	0.345	0.27
Epi_8	CD74	1.2067	0.00E+00	0.752	0.26
Epi_8	HLA-DRB1	0.8672	0.00E+00	0.527	0.119
Epi_8	HLA-DPA1	0.6175	2.85E-251	0.412	0.092
Epi_8	KRT23	1.4908	1.78E-249	0.742	0.312
Epi_8	PDZK1IP1	0.6802	6.18E-241	0.464	0.12
Epi_8	HLA-DRA	1.0005	5.28E-240	0.535	0.159
Epi_8	FXYD3	0.8534	2.27E-218	0.929	0.594
Epi_8	CRABP2	0.9997	1.14E-214	0.592	0.218
Epi_8	NAPRT	0.7518	1.10E-209	0.772	0.364
Epi_8	CST3	0.8053	6.83E-209	0.984	0.814
Epi_8	KRT20	1.0423	1.63E-198	0.921	0.64
Epi_8	KRT18	0.7239	7.11E-191	0.988	0.959
Epi_8	S100P	1.1126	1.15E-190	0.547	0.188
Epi_8	SYNGR2	0.6881	2.52E-189	0.966	0.727
Epi_8	C15orf48	0.9388	5.04E-186	0.825	0.414
Epi_8	KRT19	0.6256	9.91E-186	0.989	0.927
Epi_8	UPK2	0.8840	8.53E-170	0.803	0.426
Epi_8	CSTB	0.9192	3.58E-167	0.993	0.898
Epi_8	IFITM1	0.6745	5.68E-164	0.628	0.257
Epi_8	IL18	0.7999	1.46E-155	0.88	0.601
Epi_8	PYGB	0.6133	4.77E-154	0.812	0.466
Epi_8	SQRDL	0.5358	6.79E-154	0.647	0.301
Epi_8	LY6D	0.9774	3.65E-152	0.89	0.561
Epi_8	MMP7	1.1269	1.59E-151	0.92	0.701
Epi_8	NNMT	1.0668	4.44E-149	0.739	0.41
Epi_8	TACSTD2	0.6724	2.00E-137	0.965	0.732
Epi_8	PHLDA3	0.6480	3.26E-137	0.79	0.466
Epi_8	GSTK1	0.6005	5.39E-137	0.878	0.584
Epi_8	GPRC5A	0.5435	1.74E-136	0.967	0.771
Epi_8	RARRES3	0.8375	4.34E-135	0.769	0.421
Epi_8	CYBA	0.5865	9.15E-129	0.95	0.695
Epi_8	GLRX	0.6093	4.48E-126	0.832	0.506
Epi_8	KLK7	0.6228	1.06E-125	0.799	0.461
Epi_8	LY6E	0.5648	1.55E-123	0.976	0.796
Epi_8	CLDN3	0.6126	1.47E-121	0.955	0.735
Epi_8	S100A14	0.6373	1.63E-115	0.942	0.706
Epi_8	KRT8	0.5499	8.80E-113	0.988	0.957
Epi_8	CTSZ	0.4988	2.36E-111	0.698	0.383
Epi_8	GDF15	0.6135	1.05E-109	0.698	0.364
Epi_8	TSPAN1	0.5433	9.82E-108	0.896	0.615
Epi_8	HLA-B	0.5958	5.21E-106	0.962	0.719
Epi_8	IFI6	0.6865	4.15E-102	0.896	0.599
Epi_8	CLIC3	0.5117	1.38E-89	0.811	0.498
Epi_8	C9orf16	0.5312	1.19E-85	0.963	0.766

cluster	gene	log2_avgFC	p_val_adj	pct.1	pct.2
Epi_8	LCN2	0.5867	5.59E-74	0.949	0.759
Epi_8	CKB	0.5881	6.59E-71	0.885	0.62
Epi_8	TIMP1	0.5298	1.04E-69	0.856	0.592
Epi_8	CD24	0.5548	1.80E-66	0.888	0.671
Epi_8	EDN1	0.5620	3.47E-59	0.621	0.383
Epi_8	FTL	0.5696	2.51E-27	0.998	0.938
Epi_9	MMP3	2.8318	0.00E+00	0.764	0.123
Epi_9	TSPAN8	2.3435	0.00E+00	0.819	0.151
Epi_9	AC006262.5	1.6861	0.00E+00	0.753	0.139
Epi_9	SPINK1	2.0406	6.36E-290	0.771	0.158
Epi_9	FTH1	2.0462	2.93E-242	1	0.988
Epi_9	MMP12	0.9593	8.56E-197	0.283	0.026
Epi_9	HLA-A	1.7242	1.34E-192	1	0.803
Epi_9	HLA-B	1.8590	8.61E-185	0.984	0.73
Epi_9	B2M	1.4093	8.61E-169	1	0.909
Epi_9	HPGD	1.8103	3.03E-165	0.961	0.633
Epi_9	CEACAM6	1.1783	3.94E-164	0.413	0.069
Epi_9	PERP	1.2678	8.22E-164	0.984	0.841
Epi_9	CST3	1.7680	6.67E-161	0.993	0.821
Epi_9	HLA-C	1.5152	3.78E-145	0.961	0.72
Epi_9	RP11-297P16.4	1.3326	4.06E-144	0.703	0.236
Epi_9	C15orf48	1.7445	1.61E-141	0.887	0.432
Epi_9	YPEL3	1.1569	1.52E-140	0.692	0.229
Epi_9	TPT1	0.9989	1.64E-136	0.993	0.89
Epi_9	PPDPF	1.2976	6.04E-131	0.971	0.783
Epi_9	PDZK1IP1	1.0092	6.32E-131	0.531	0.135
Epi_9	PRSS3	1.0623	1.53E-129	0.669	0.228
Epi_9	SECTM1	1.0304	1.31E-127	0.558	0.161
Epi_9	CAMK2N1	1.5277	4.54E-126	0.9	0.572
Epi_9	FXYD3	1.4384	2.12E-124	0.923	0.61
Epi_9	NEAT1	1.2845	2.29E-120	0.971	0.686
Epi_9	CD24	1.4473	1.39E-110	0.943	0.68
Epi_9	MALAT1	0.9077	4.38E-107	1	0.9
Epi_9	FTL	0.8843	9.40E-101	0.998	0.94
Epi_9	PLAT	1.2909	6.30E-100	0.603	0.211
Epi_9	PRSS8	1.0668	5.30E-98	0.712	0.33
Epi_9	CD82	1.0289	8.73E-98	0.628	0.245
Epi_9	CDA	1.0058	6.01E-96	0.873	0.65
Epi_9	SULT2B1	0.9302	8.82E-96	0.646	0.253
Epi_9	CITED2	1.2036	3.96E-93	0.692	0.304
Epi_9	KRT16	1.2962	1.96E-90	0.628	0.246
Epi_9	FXYD5	0.8947	4.20E-88	0.927	0.722
Epi_9	LIPH	1.1674	1.61E-87	0.707	0.323
Epi_9	GABARAPL1	0.8812	1.63E-87	0.522	0.173
Epi_9	GSTK1	1.0699	1.13E-86	0.859	0.599
Epi_9	SERINC2	0.9834	3.10E-81	0.834	0.566
Epi_9	PRSS22	1.1629	2.65E-64	0.841	0.567
Epi_9	ADIRF	0.9410	5.52E-63	0.984	0.849
Epi_9	CTSD	0.9664	3.92E-57	0.912	0.723
Epi_9	IFI6	1.2831	2.46E-55	0.834	0.615

cluster	gene	log2_avgFC	p_val_adj	pct.1	pct.2
Epi_9	LCN2	1.1184	1.46E-53	0.98	0.767
Epi_9	KRT17	1.0370	3.19E-48	0.85	0.584
Epi_9	GDF15	1.3826	1.04E-46	0.644	0.381
Epi_9	KLK10	0.9241	5.41E-33	0.664	0.466
Epi_9	SPRR3	1.0420	5.41E-28	0.363	0.16
Epi_9	UPK2	0.9392	1.52E-27	0.63	0.448
Epi_10	SNHG12	2.6315	0.00E+00	0.659	0.094
Epi_10	DDIT3	2.6618	1.18E-271	0.735	0.151
Epi_10	EPB41L4A-AS1	2.1125	1.66E-190	0.713	0.21
Epi_10	SNHG15	2.4521	2.82E-185	0.832	0.354
Epi_10	RSRC2	2.1336	6.21E-170	0.832	0.352
Epi_10	TAF1D	2.5748	2.54E-168	0.878	0.523
Epi_10	TXNIP	2.2981	1.07E-164	0.599	0.15
Epi_10	CYR61	2.2149	5.92E-144	0.453	0.09
Epi_10	HIST1H4H	1.5048	1.45E-127	0.263	0.033
Epi_10	ATF3	2.2331	2.87E-125	0.642	0.224
Epi_10	GADD45B	2.7686	3.17E-124	0.783	0.419
Epi_10	SNHG8	2.4073	1.58E-122	0.735	0.357
Epi_10	RBM39	1.8177	2.81E-121	0.878	0.664
Epi_10	ZFAS1	2.7501	1.08E-119	0.849	0.702
Epi_10	BRD2	1.7365	9.68E-116	0.745	0.348
Epi_10	PPP1R15A	2.1387	7.94E-115	0.83	0.526
Epi_10	SLC3A2	2.1405	4.35E-113	0.793	0.511
Epi_10	BIRC3	2.1977	2.75E-101	0.533	0.171
Epi_10	ATF4	1.7481	6.99E-100	0.798	0.476
Epi_10	CWC25	1.4870	2.23E-98	0.625	0.24
Epi_10	C6orf48	2.1230	4.07E-93	0.745	0.467
Epi_10	TRIB3	2.0802	1.15E-88	0.547	0.202
Epi_10	ZNF830	1.5164	8.11E-86	0.467	0.143
Epi_10	HIST1H2BG	2.4298	2.92E-84	0.365	0.09
Epi_10	MYC	1.6414	4.25E-79	0.484	0.169
Epi_10	HIST1H2AC	2.0745	2.21E-77	0.487	0.172
Epi_10	LUC7L3	1.4129	3.68E-77	0.74	0.418
Epi_10	NR1D1	1.8499	4.06E-75	0.543	0.216
Epi_10	SNHG19	1.4528	7.84E-72	0.394	0.117
Epi_10	CEBPB	1.7818	8.56E-72	0.691	0.448
Epi_10	SNHG7	1.6294	8.71E-71	0.713	0.466
Epi_10	OSER1	1.5709	1.41E-69	0.584	0.271
Epi_10	UPP1	1.5143	9.65E-69	0.499	0.195
Epi_10	BTG1	1.4772	6.23E-68	0.659	0.366
Epi_10	GADD45A	2.4229	2.17E-63	0.696	0.497
Epi_10	RPL22L1	1.5085	1.18E-61	0.725	0.462
Epi_10	CTA-29F11.1	1.4387	1.54E-56	0.409	0.145
Epi_10	HIST1H1C	2.5761	3.58E-56	0.589	0.318
Epi_10	HIST3H2A	1.7935	3.89E-55	0.45	0.186
Epi_10	ODC1	1.5014	7.18E-51	0.681	0.484
Epi_10	HIST1H2BD	1.5689	8.73E-46	0.372	0.139
Epi_10	DDIT4	1.4738	6.46E-42	0.477	0.227
Epi_10	CITED2	1.8845	2.52E-41	0.555	0.308
Epi_10	HIST1H2AE	1.4278	4.06E-37	0.277	0.092

cluster	gene	log2_avgFC	p_val_adj	pct.1	pct.2
Epi_10	ARRDC3	1.6578	2.88E-34	0.314	0.121
Epi_10	PMAIP1	1.6405	6.84E-29	0.655	0.514
Epi_10	G0S2	1.8180	6.79E-22	0.555	0.43
Epi_10	KRTAP2-3	1.5666	3.39E-04	0.28	0.192
Epi_10	KRTAP3-1	2.4622	1.00E+00	0.277	0.235
Epi_10	HES1	1.5499	1.00E+00	0.516	0.64
Epi_11	TUBA1A	2.3948	0.00E+00	0.921	0.078
Epi_11	IGFBP7	2.2486	0.00E+00	0.917	0.042
Epi_11	PRSS33	2.0595	0.00E+00	0.929	0.059
Epi_11	EPHB6	2.0301	0.00E+00	0.896	0.106
Epi_11	SLC14A1	2.0111	0.00E+00	0.946	0.07
Epi_11	PRF1	1.8569	0.00E+00	0.855	0.017
Epi_11	BLVRA	1.7379	8.37E-198	0.896	0.21
Epi_11	SERPINE1	1.3931	9.60E-148	0.801	0.187
Epi_11	EMP3	1.6599	1.32E-141	0.971	0.394
Epi_11	IGFBP6	2.2625	9.15E-135	0.988	0.492
Epi_11	TPM2	1.0975	6.39E-133	0.739	0.17
Epi_11	PPIA	1.9360	1.16E-121	1	0.829
Epi_11	NPC2	1.9213	5.27E-118	0.992	0.575
Epi_11	STK17A	1.0388	1.98E-106	0.701	0.187
Epi_11	CCM2	1.1150	1.26E-104	0.846	0.295
Epi_11	NT5E	1.3800	1.48E-103	0.9	0.389
Epi_11	UPP1	0.9571	3.95E-103	0.722	0.195
Epi_11	SH3BGRL3	1.2813	6.36E-101	1	0.85
Epi_11	ANXA5	1.0826	1.44E-99	0.834	0.306
Epi_11	ATOX1	1.3561	4.10E-99	0.95	0.511
Epi_11	IFI27L2	1.0578	1.49E-95	0.83	0.308
Epi_11	TMED4	1.3434	2.05E-95	0.859	0.355
Epi_11	TGFBI	1.2851	4.82E-94	0.95	0.499
Epi_11	TIMP1	1.2644	1.72E-93	0.979	0.606
Epi_11	IFI27	1.2053	4.52E-93	1	0.861
Epi_11	CD74	1.2010	1.25E-90	0.851	0.288
Epi_11	S100A6	1.1570	3.93E-89	1	0.969
Epi_11	DUSP6	1.4211	9.19E-89	0.959	0.528
Epi_11	STMN1	1.3378	2.22E-88	0.979	0.606
Epi_11	NDUFB2	1.2295	2.05E-87	0.988	0.749
Epi_11	PPP3CA	1.0392	1.40E-82	0.693	0.208
Epi_11	LGALS1	1.3434	1.87E-82	0.996	0.75
Epi_11	NPW	1.2271	3.77E-81	0.971	0.524
Epi_11	PSMA2	1.2667	1.59E-79	0.9	0.506
Epi_11	MET	1.1755	1.81E-78	0.959	0.604
Epi_11	PTMS	0.9962	7.60E-74	0.992	0.696
Epi_11	PLK2	1.0595	9.37E-74	0.867	0.384
Epi_11	POLD2	1.1827	1.87E-73	0.905	0.468
Epi_11	RPL22L1	1.2351	4.67E-70	0.876	0.463
Epi_11	TES	0.9738	4.16E-69	0.851	0.39
Epi_11	ANKRD1	1.0353	1.41E-67	0.419	0.09
Epi_11	MT-ND3	1.1342	4.41E-66	1	0.747
Epi_11	H2AFV	1.4779	1.16E-57	0.863	0.518
Epi_11	AREG	1.1939	7.51E-56	0.975	0.7
Epi_11	ISG15	1.0550	2.41E-54	0.988	0.727

cluster	gene	log2_avgFC	p_val_adj	pct.1	pct.2
Epi_11	RHOBTB3	1.0621	1.73E-50	0.801	0.41
Epi_11	MDK	1.0745	1.98E-46	0.805	0.417
Epi_11	CAV1	1.1569	1.36E-44	0.983	0.712
Epi_11	IGFBP1	1.2287	2.93E-44	0.606	0.241
Epi_11	CAV2	0.9671	1.57E-39	0.921	0.648
